# Spine endoscopic surgery establishment for disc disease (Neurocore-SENSED): an open and decentralized consensus

**DOI:** 10.1016/j.bas.2025.105604

**Published:** 2025-09-13

**Authors:** Mejdeddine Al Barajraji, Sami Barrit, Adam Boukind, Albert E. Telfeian, Javier Quillo-Olvera, Mehdi Afathi, Frank Hassel, Peter B. Derman, Maxime Challali, Alexandre Simonin, Antoine Devalckeneer, Ryoji Tominaga, Jean-Charles Le Huec, Xavier A. Santander, Stefan Motov, Richard Assaker, Thibault Remacle, Neurocore-SENSED Research Group, Khaled Radjouane, Brenda Bueno, Katsuhiko Ishibashi, Mohamed Dehmani, Mohamed Arnaout, Raed Abohussein, Kern Singh, Gregory Kesteloot, Mehmet Zileli, Puneet Bansal, Osama Kashlan, Martin Caudron, Maximo De Zavalia, Hisco Robjin, Fah Bouare, Ricky Rasschaert, Nicolas Gil Guevara, Nikhil Jain, Matthieu Vassal, Nikolaos Haliasos, Abdulhamid Ciçek, M.D. Henri d’Astrog, Maxime Challali, Arnaud Lomdard, Grish Datar, Peter Van Daele, Hieu Kim Huynh, Dieter Thijs, Leonardo Tariciotti, Jean Destandau, Frederic Jacquot, Hadrien Giorgi, Marcelo Amato, Loïc de Nijs, Triantafullos Bouras, Constantine Constantoyannis, Alfonso Garcia, Marta Garvayo, Francois Gerardy, Boulos Ghannam, Herve Monka Lekuya, Krzystof Suszynski, Dimitri Vanhauwaert, Martin Dupuy, Karim Chirani, Stephanie Nunes Da Paz, Vincent Bex

**Affiliations:** w Department of Neurosurgery, Military Hospital of Tunis, Tunisia; x Cliniques Universitaires Saint-Luc – Neurochirurgie, Saint-Luc, Switzerland; y Iwai Orthopaedic Hospital, Edogawa, Tokyo, Japan; z Neurosurgery Department, Faculty of Medicine, Zagazig University, Sharkia, Zagazig, Egypt; aa Sankt Elisabeth Hospital, Gütersloh, Germany; ab Rush University Medical Center, Midwest Orthopaedics at Rush, US; ac AZ Monica Antwerpen, Belgium; ad Ege University Neurosurgery Department, Izmir, Türkiye; ae Alaji Cure & Care Hospital Jaipur, India; af Weill Cornell Medicine, New York City, New York, USA; ag CClinique Saint-Pierre Ottignies, Ottignies-Louvain-la-Neuve, Belgium; ah CEMIC, Buenos Aires, Argentina; ai Regional Hospital of Tienen, Tienen, Belgium; aj King Mohammed VI University Teaching Hospital, Marrakesh, Morocco; ak AZ Rivierenland, Department of Neurosurgery, Belgium; al National Cancer Institute / Clinica del Country - Clinica La Colina / Clinica del Occidente, Columbia, USA; am Manipal Hospitals, Dwarka, New Delhi, India; an Clinique Saint Jean Sud de France, Saint Jean de védas, France; ao Department of Neurosurgery, Metropolitan Hospital, Athens, Greece; ap Neurosurgery, ZOL, Genk, Belgium; aq Centre Orthopédique Santy, Lyon, France; ar Clinic du palais France, Grasse, France; as Centre for Economic Spine Surgery, Sushruta Hospital for Orthopedics & Traumatology, Hospital Road, Behind Hotel arafa, Miraj, (MH), 41741, India; at Ziekenhuis Waregem, O.L.V van Lourdes Ziekenhuis Waregem, Belgium; au Can Tho University of Medicine and Pharmacy, Ninh Kiều, Cần Thơ, Viet Nam; av Department of Neurosurgery, Antwerp University Hospital, Edegem, Belgium; aw University of Milan, Milan, Italy; ax Centre Tourville, 17 av de Tourville, 75007, Paris, France; ay Institut Méditerranéen du Dos, Marseille, France; az University of São Paulo - Medical School of Ribeirão Preto, Brazil; ba Cliniques Universitaires Saint-Luc – Neurochirurgie, Brussels, Belgium; bb Department of Neurosurgery, CHWAPI, Tournai, Belgium; bc Department of Neurosurgery, Patras University Hospital, Patras, Greece; bd Endoscopic Spine Surgery / Hospital Angeles Tijuana, Mexico; be Muhimbili Orthopeadic Institute (MOI), Dar es Salaam, Tanzania in Collaboration with Weill Cornell Medicine, NY, USA; bf CHU de Lille, Lille, France; bg Makerere University, Kampala, Uganda; bh Sporta Medicine and phisiology of physical effort Depot., Medical University of Silesia in Katowice, Poland; bi AZ Delta Roeselare, Belgium; bj Neurosurgery Department Clinique de l'Union, Toulouse, France; bk Neurosurgery Department, Henri, Mondor University Hospital, Créteil, France; bl Department of Neurosurgery, CHR Citadelle, Liege, Bd du Douzième de Ligne 1, 4000, Liège, Belgium; bm Bordeaux Nord Aquitaine Polyclinique, 3 Rue du Dr Finlay, 33300, Bordeaux, France; a Department of Neurosurgery, CHR Citadelle, Liege, Bd du Douzième de Ligne 1, 4000, Liège, Belgium; b Faculty of Medicine, UMONS Research Institute for Health Sciences and Technology, University of Mons (UMONS), 8 avenue du Champ de Mars, 7000, Mons, Belgium; c Department of Neurological Surgery, CHU Tivoli ULB, Avenue Max Buset, 34, 7110, La Louvière, Belgium; d Sciense, New York, NY, 10013, USA; e Institut de Neurosciences des Systèmes, Aix-Marseille Université, 27 Bd Jean Moulin, 13005, Marseille, France; f Department of Neurological Surgery, University of California, 400 Parnassus Ave, 8th Floor, San Francisco, CA, 94143, USA; g Computational Precision Health, University of California, Berkeley, CA, USA; h Division of Plastic Surgery, Department of Surgery, Washington University School of Medicine, 4921 Parkview PI#6G, St. Louis, MO, 63110, USA; i Neurosurgery Department, Brown University, 593 Eddy St, Providence, RI, USA; j Spine Center - Neurological Surgery Department, Hospital Angeles Centro Sur, Boulevard Bernado Quintana Arrioja 9670, Querétaro, Mexico; k Clinique Saint-Charles, 25 Rue de Flesselles, Lyon, France; l Departement of Spine Surgery, Loretto-Krankenhaus Freiburg, 79100, Freiburg im Breisgau, Germany; m Texas Back Institute, 12222 N Central Expy Suite 310, Dallas, TX, 75243, USA; n Clinic du Palais, 25 Av. Chiris, Grasse, 06130, France; o Department of Neurosurgery, Hôpital de Sion, Av. du Grand-Champsec 80, 1951, Sion, Switzerland; p Centre Hospitalier Universitaire de Lille, 2 Av. Oscar Lambret, 59000, Lille, France; q Iwai Medical Foundation Iwai Orthopedic Medical Hospital, 8-chōme-17-2 Minamikoiwa, Edogawa City, Tokyo, Japan; r Iwai FESS Clinic, Edogawa, 8-18-4 Minamikoiwa, Edogawa-ku, Tokyo, 133-0056, Japan; s Bordeaux Nord Aquitaine Polyclinique, 3 Rue du Dr Finlay, 33300, Bordeaux, France; t Instituto Clavel, C. de Joaquín Costa, 28, primera planta, Chamartín, 28002, Madrid, Spain; u Spine Center of Eastern Switzerland & Department of Neurosurgery, Rorschacher Str. 95/Haus 07, Kantonsspital St. Gallen, 9007 St, Gallen, Switzerland; v CHRU Lille, Hôpital Salengro, Rue Emile Laine, 59037, Lille, France

**Keywords:** Delphi technique, Endoscopic spine surgery, Minimally invasive, Consensus, Disc diseases, Standardization, Intervertebral disc, Spine endoscopy

## Abstract

**Introduction:**

Endoscopic spine surgery (ESS) has evolved considerably, yet there remains a lack of consensus on patient selection, technical specifications, and outcome reporting. This heterogeneity hinders collaborative efforts to establish comprehensive evidence-based practices.

**Research question:**

To develop core outcome sets (COS) and reporting guidelines for ESS for disc disease through an international consensus.

**Material and methods:**

We established a multidisciplinary, international expert panel through literature review and peer-to-peer recruitment. Between January and April 2025, we employed a three-round modified Delphi technique. Items spanned three domains: patient characteristics, practices and outcomes. Propositions endorsed by ≥ 75 % of participants were classified as 'strong' and included in the COS. Those with 50–75 % were reconsidered in the subsequent round, requiring ≥66 % for inclusion as 'moderate'.

**Results:**

The panel consisted of 77 spine surgeons from Europe (61 %), Asia (14 %), and North America (13 %), with 58 completing all three rounds. Of 183 items (165 initially provided, 18 participant add-ons), agreement was reached on 99 items (82 strongly and 17 moderately). Key consensus elements included demographics, comorbidities, clinical complaints, imaging findings, surgical indications, technical parameters, and outcome measures such as pain scores, functional outcomes, and complications.

**Discussion and conclusion:**

We established the Neurocore-SENSED framework, comprising COS and reporting guidelines for ESS for disc disease. This consensus addresses fundamental heterogeneity in ESS research, complementing existing nomenclature and indication-specific guidelines. By enhancing consistency, comparability, and quality of ESS research, this framework aims to accelerate the development of evidence-based practices that optimize patient outcomes in this rapidly advancing field.

## Highlights

• Defined core outcome sets (COS) and reporting guidelines for endoscopic spine surgery (ESS) for disc disease through an international, multidisciplinary consensus process.
• Delphi consensus (3 rounds) process involving 77 spine surgeons.
• Reached consensus on 99 key items across patient, practice, and outcomes domains.
• By enhancing consistency, comparability, and quality of ESS research, this framework aims to accelerate the development of evidence-based practices that optimize patient outcomes in a rapidly advancing field.

## 1 Introduction

Endoscopic spine surgery (ESS) has evolved considerably over the past decades, progressing from early percutaneous techniques introduced in the 1970s (Hijikata, 1989) to sophisticated minimally invasive procedures that are increasingly recognized in modern spine care (Burkett and Brooks, 2024; Jang et al., 2021; Tang et al., 2023). Technological advancements in optics, instrumentation, and surgical approaches have enabled the development of various endoscopic techniques—including uniportal and biportal endoscopic procedures for cervical, thoracic, and lumbar regions. With indications ranging from the treatment of degenerative diseases such as herniated discs to the decompression of neural elements in complex anatomical situations (Tang et al., 2023; Simpson et al., 2022).

Evidence indicates that while ESS offers significant advantages, such as minimized tissue trauma, reduced blood loss, lower infection rate, shorter hospital stay (with a 30-day reduced readmission rate compared with nonendoscopic surgeries), and quicker return to daily activities, it may not be universally applicable to all spinal pathologies (Burkett and Brooks, 2024; Ahn, 2019; Butler et al., 2019; Chen et al., 2023; Compagnone et al., 2024; Leyendecker et al., 2025). For example, while the high-quality evidence for transforaminal lumbar discectomy is compelling, the data for more complex procedures, such as endoscopic decompression for severe spinal stenosis or tumor resection, are still emerging (Hasan et al., 2019; Suvithayasiri et al., 2023; Ali et al., 2022)–(Hasan et al., 2019; Suvithayasiri et al., 2023; Ali et al., 2022).

Despite advances in established nomenclature (Hofstetter et al., 2020) and indication-specific guidelines (Farshad et al., 2025; Davies et al., 2024; Deer et al., 2019), there remains a lack of an overarching consensus on patient selection, technical specifications, and outcome reporting in ESS. This heterogeneity in the literature hinders collaborative efforts to establish comprehensive evidence-based practices across the full spectrum of ESS applications. Core outcome sets (COS) and reporting guidelines represent instrumental harmonization tools that can provide structured frameworks for defining and reporting key outcomes, thereby enhancing research quality, comparability, and reproducibility (Simera et al., 2010; Webbe et al., 2018).

Here, we conducted an open, decentralized consensus study to develop COS and reporting guidelines for ESS for disc disease. This approach complements previous efforts by capturing expertise from diverse spine surgeons versed in endoscopic technique, using an inclusive and robust methodology.

## 2 Material and Methods

### 2.1 Literature review and expert panel establishment

A preliminary literature review identified existing research, consensus, and guidelines related to endoscopic spine surgery (ESS). We searched PubMed, Embase, and Cochrane databases using the following keywords: ["endoscopic spine surgery" OR "minimally invasive spine surgery" OR "ESS" OR "spine endoscopy" OR "spinal endoscopy"] AND ["disc" OR "discectomy" OR "intervertebral disc" OR "lumbar" OR "cervical" OR "thoracic" OR "dorsal"].

A multidisciplinary, international consensus panel was established through the preliminary literature review for field contributors, professional communication channels such as learned societies, and peer-to-peer recruitment. This panel was open to any qualified individuals with clinical or scientific expertise in ESS. Two co-investigators confirmed this expertise, verifying each participant's credentials using publicly available information such as institutional profiles, publication records, and author databases, while ensuring consistency with the participant's email address.

### 2.2 Delphi methodology

Between January and April 2025, we employed a three-round web-based modified Delphi technique to define a COS and reporting guidelines for ESS. In line with Delphi methodology, informed consent was implied through participation. The study was deemed exempt from institutional review board approval. None of the experts had access to the participant list, preventing any consultation before responding. An initial questionnaire, formulated based on the preliminary literature review, consisted of multiple-choice and open-ended questions. Each round of questions was distributed anonymously via email to ensure the experts were unaware of each other's identities and responses. Two investigators supervised the process.

### 2.3 Domain structure and consensus criteria

As a working basis, items were proposed in three domains: "patient characteristics" (demographics, comorbidities, initial complaints, severity assessments, and treatments), "practices" (clinical, surgical, and technical), and "outcomes" (reporting methods, complications, and adverse events). We defined ESS as minimally invasive surgical procedures performed using endoscopic techniques to access and treat spinal pathologies, focusing on disc disease.

Each section included free-text fields to capture participant-driven insights or additional propositions, termed "add-ons," which were included in subsequent rounds. Add-ons were either directly incorporated into the next round, integrated with other propositions, or considered valuable insights without forming direct propositions based on a consensus among three investigators.

Propositions endorsed by ≥ 75 % of participants were classified as 'strong agreement' and included in the COS. Those with 50–75 % endorsement were reconsidered in the subsequent round, requiring at least a pre-determined threshold of 66 % for inclusion as 'moderate agreement.' Propositions with <50 % endorsement were considered lacking consensus and therefore excluded. Participants had the opportunity to request reconsideration of any included or excluded proposition. Each proposition was subject to a maximum of two consecutive rounds of consideration, either between rounds 1 and 2 or rounds 2 and 3.

After each round, comprehensive group response feedback, along with the add-ons, were provided to the panel of participants in an "open report." To minimize response fatigue, each round's questionnaire was designed to be concise, taking less than 15 min to complete.

## 3 Results

### 3.1 Panel characteristics

The expert panel initially consisted of 77 spine surgeons primarily from Europe (61 %), Asia (14 %), and North America (13 %). Fifty-eight of them completed the three rounds for an overall drop-out rate of 25 %. Among the first-round respondents, 32 % were spine neurosurgeons, 31 % spine orthopedists, 30 % general neurosurgeons, and 7 % general orthopedists. The majority of participants were actively involved in ESS practice, with 76 % reporting performing these procedures on a daily or weekly basis, either in clinical or research roles. Over 35 % of the participants had more than five years of experience in ESS.

### 3.2 Overall consensus

Of the 183 items, 165 were initially provided, and 18 (9.8 %) were derived from add-ons. Agreement was reached on 99 items, with 82 achieving strong and 17 moderate levels. The distribution of items and sub-items, including add-ons, is summarized in Table 1 and detailed in Appendix A.

**Table 1:** Overall distribution of consensus results by domain.

Domain	Total items	Strong agreement (≥75 %)	Moderate agreement (66–74 %)	No agreement (<66 %)
Patient characteristics	65	30 (46.2 %)	8 (12.3 %)	27 (41.5 %)
Practices	86	32 (37.2 %)	7 (8.1 %)	47 (54.7 %)
Outcomes	32	20 (62.5 %)	2 (6.2 %)	10 (31.2 %)
**Total**	**183**	**82 (44.8 %)**	**17 (9.3 %)**	**84 (45.9 %)**

### 3.3 Patient characteristics

Overall, 27 items (41.5 %) did not reach agreement, 8 items (12.3 %) achieved moderate agreement, and 30 items (46.2 %) reached strong agreement (key items are presented in Table 2, all items are provided in Appendix B).

**Table 2:** Patient characteristics domain.

Subdomain	Strong agreement items (≥75 %)	Agreement
Demographics	Age	99 %
	Body mass index	96 %
	Sex/gender	91 %
	Occupation	86 %
Comorbidities	Spine surgery history	97 %
	Diabetes	87 %
	Coagulation/platelet disorders	84 %
	Neurological conditions	78 %
Initial complaints	Pain characteristics	99 %
	Radicular symptoms	98 %
	Limb weakness	96 %
	Numbness or tingling	94 %
Severity assessment	Direction of disc herniation	92 %
	Disc height	86 %
	Modic changes	84 %
	VAS pain scoring	82 %
	ODI	80 %

Age (99 %), body mass index (96 %), and sex/gender (91 %) were the most selected demographic variables in patient profiling. Occupation (86 %) was also deemed important.

Comorbidities were considered important for reporting in research, with spine surgery history (97 %), diabetes (87 %), coagulation/platelet disorders (84 %), and neurological conditions (78 %) being the most frequently chosen.

For initial patient complaints, pain characteristics (99 %), radicular symptoms (98 %), limb weakness (96 %), numbness or tingling (94 %), difficulty walking or standing (87 %), and bowel or bladder dysfunction (87 %) all reached strong agreement.

For severity assessment, both imaging-based morphological criteria (79 %) and validated clinical scoring systems (79 %) were prioritized. Among morphological severity criteria, direction of disc herniation ((para)central, (extra)foraminal) (92 %), disc height (86 %), Modic changes (84 %), Meyerding grading (83 %), and type of disc herniation (79 %) reached strong agreement. For clinical severity scoring systems, the Visual Analog Scale (VAS) pain scoring system (82 %) and the Oswestry Disability Index (ODI) (80 %) were the preferred assessments.

Among active treatments, opioids (83 %), neuropathic pain medications (79 %), corticosteroids (79 %), anticoagulants/antithrombotics (78 %), and nonsteroidal anti-inflammatory drugs (NSAIDs) (78 %) were prioritized for reporting.

### 3.4 Practices

Overall, 47 items (54.7 %) did not reach agreement, 7 items (8.1 %) achieved moderate agreement, and 32 items (37.2 %) reached strong agreement (key items are presented in Table 3, all items are provided in Appendix C).

**Table 3:** Practices domain.

Subdomain	Strong agreement items (≥75 %)	Agreement
Clinical practice	Duration of symptoms before surgery	96 %
	Steroid injections (conservative therapy)	91 %
	Conservative therapy trial prior to surgery	86 %
	Physical therapy	83 %
	Prior same-level herniation	83 %
Surgical practice	Specific spinal level(s) treated	92 %
	Surgical approach	92 %
	Indication for surgery	91 %
	Duration of surgery	88 %
	Uniportal vs biportal technique	87 %
Technical details	Pump type and settings for irrigation	92 %
	Drill type	91 %
	Radiofrequency/laser purpose	87 %
	Drill diameter	80 %

In clinical practice, duration of symptoms before surgery (96 %) and conservative therapy trial prior to surgery (86 %) were highly prioritized in patient care protocols. Conservative therapy with steroid injections (91 %) and physical therapy (83 %) reached strong agreement. Prior occurrence of same-level herniation (83 %) were also considered important to report.

In surgical practice, specific spinal level(s) treated (92 %), surgical approach (92 %), and indication for surgery (91 %) received the highest agreement. All specific indications for surgery (severe intractable pain, radicular pain with functional impairment, progressive neurological deficits, failed conservative treatment, cauda equina syndrome, and recurrent disc herniation) achieved strong agreement (81–90 %). Duration of surgery (88 %), with estimation in minutes (88 %), and if uniportal or biportal technique was used (87 %) were also deemed crucial for reporting.

Technical details related to ESS reached moderate agreement for several categories. Within these categories, specific sub-items such as pump type and settings for irrigation (92 %), drill type (91 %), and drill diameter (80 %) reached strong agreement. Similarly, for radiofrequency or laser use, purpose (87 %) and settings (76 %) showed strong agreement.

### 3.5 Outcomes

Overall, 10 items (31.2 %) did not reach agreement, 2 items (6.2 %) achieved moderate agreement, and 20 items (62.5 %) reached strong agreement (key items are presented in Table 4, all items are provided in Appendix D).

**Table 4:** Outcomes domain.

Subdomain	Strong agreement items (≥75 %)	Agreement
Outcome indicators	NRS pain scoring	100 %
	Systematic outcome reporting	97 %
	Hospital stay duration	91 %
	Return to work/daily activities	90 %
	MRC grading (motor function)	90 %
	VAS pain scoring	80 %
	ODI	79 %
Complications	Systematic complication reporting	100 %
	Persistent or worsened pain	97 %
	Incidental durotomy	94 %
	Nerve injury	94 %
	Conversion to open surgery	91 %
	Recurrent disc herniation	88 %
	Postoperative hematoma	87 %
	Return to OR within 30 days	86 %

Systematic outcome reporting was strongly supported (97 %). Among outcome indicators, strong agreement was achieved for reporting pain using both the NRS (100 %) and the VAS (80 %), motor neurological function via MRC grading (90 %), functional outcomes via hospital stay duration (91 %), return to work or daily activities (90 %) and the ODI (79 %). Reporting of complication (81 %) and reoperation (76 %) rates was considered a high priority.

There was unanimous consensus that complications should be reported systematically (100 %). Among specific complications, the highest levels of consensus were observed for persistent or worsened pain (97 %), incidental durotomy and nerve injury (both 94 %), and failure to complete the procedure endoscopically requiring conversion (91 %). Strong agreement was also reached for postoperative hematoma (87 %), recurrent disc herniation (88 %), returning to the operating room within 30 days (86 %), neuropathic pain (84 %), cerebrospinal fluid (CSF) leak (83 %) and postoperative surgical site infection (81 %). Moderate agreement was found for vascular injury (67 %). Wrong-site surgery and injury to major structures did not achieve consensus (respectively 64 % and 48 %).

## 4 Discussion

This open, decentralized, three-round Delphi study involved 77 expert spine surgeons and researchers. We established the Neurocore-SENSED framework, comprising COS and reporting guidelines for ESS for disc disease. Of 183 items, agreement was reached on 99 items, spanning the three domains of patient characteristics, practices, and reported outcomes.

### 4.1 Key findings and clinical implications

Beyond basic demographic variables, we identified critical baseline attributes for consistent patient profiling in ESS. Age, body mass index, sex/gender, and occupation achieved strong agreement as key demographic variables. These factors are critical for risk stratification and understanding outcomes variations in spine surgery, including ESS(Ayhan et al., 2018; Hébert et al., 2020). The strong consensus on spine surgery history, diabetes, coagulation disorders, and neurological conditions reflects their significant impact on surgical decision-making and postoperative outcomes (Ono et al., 2022; Kwon and Park, 2023).

For initial patient complaints, near-unanimous agreement was achieved on reporting pain characteristics, radicular symptoms, limb weakness, and paresthesia, providing essential baseline data for evaluating treatment effectiveness. The panel equally endorsed both morphological assessment and clinical scoring systems for evaluating disc disease severity, with VAS pain scoring and ODI emerging as the preferred validated instruments.

Strong consensus on documenting symptom duration, conservative therapy trials, and surgical indications emphasizes the importance of capturing the full clinical context. The high agreement across all specific surgical indications demonstrates emerging standardization in determining appropriate candidates for ESS. The strong consensus on reporting operative duration and technique employed recognizes these parameters as key technical benchmarks that influence patient outcomes.

Technical reporting received variable agreement, with experts prioritizing parameters directly impacting surgical precision and safety over general equipment specifications. This pattern suggests that ESS has reached a stage where standardized outcome reporting and implementation guidelines are more critical than technical standardization (Kim et al., 2025; Yuh et al., 2023).

The near-unanimous support for systematic outcome reporting reflects recognition of the critical importance of structured assessment. Strong consensus on pain assessment (NRS and VAS), neurological motor function, and functional restoration highlights a pragmatic focus on patient-centered outcomes that directly reflect symptomatic improvement rather than surrogate markers (Schmid et al., 2009; Boden et al., 2020; Balza and Palmer, 2023).

The unanimous agreement on systematic complication reporting, with strong consensus on specific adverse events, emphasizes the importance of transparently documenting negative outcomes. This approach aligns with contemporary emphasis on patient-centered outcome measurement in spine surgery research (Bydon and Dominari, 2025; Beighley et al., 2022).

### 4.2 Perspectives

Our study employed established Delphi methodology with predefined consensus thresholds consistent with international standards. The 75 % threshold for strong agreement and 66 % for moderate agreement align with the median thresholds reported in systematic reviews of Delphi studies (Diamond et al., 2014). The use of descriptive statistics (percentages, means, standard deviations) for consensus measurement follows established practice in Delphi methodology, where the goal is consensus measurement rather than hypothesis testing (Holey et al., 2007; Kirkham et al., 2017).

The initial focus on surgeon-driven consensus represents a strategic response to the field's immediate needs for technical and clinical parameter standardization. While broader stakeholder involvement holds value for comprehensive healthcare initiatives, establishing fundamental parameters first creates a necessary foundation for ESS standardization that subsequent, more inclusive consensus efforts can build upon.

The Neurocore-SENSED framework complements existing society-led initiatives such as AOSpine's nomenclature consensus (Hofstetter et al., 2020) and MIST recommendations (Deer et al., 2019) by addressing the fundamental heterogeneity that has impeded collaborative research. Rather than duplicating existing guidelines, this framework provides the missing link of comprehensive outcome reporting standards that can be applied across different ESS applications and practice settings.

### 4.3 Limitations

Several limitations warrant acknowledgment. First, the expert panel was restricted to spine surgeons, which may not fully capture societal and patient-centered priorities. Second, geographical representation was uneven despite active outreach efforts, with participation remaining largely Western. Third, focusing on disc disease excludes other important ESS applications. Finally, while the Delphi method is well-established for developing consensus, it inherently reflects expert opinion rather than empirical evidence (Gargon et al., 2019; Prinsen et al., 2014).

### 4.4 Future directions

The Neurocore-SENSED framework establishes a foundation for developing more comprehensive approaches to ESS. Prospective implementation studies will validate its practical utility, while future iterations should expand to address different spinal regions and broaden stakeholder involvement. Integration with global spine registries will create a standardized data ecosystem supporting meaningful benchmarking and multicenter collaboration (Saravi et al., 2022; Ju and Lee, 2023).

## 5 Conclusions

Through an open and decentralized modified Delphi process, we established international expert agreement on critical parameters across patient characteristics, practices, and outcome domains, providing a foundation for harmonizing both research methodologies and clinical practice in ESS. This consensus complements existing society-led nomenclature and indication-specific guidelines by addressing the fundamental heterogeneity that has long impeded collaborative research and evidence-based practice in the field.

The Neurocore-SENSED framework represents a significant step toward standardizing ESS research and practice. By enhancing the consistency, comparability, and quality of ESS research, this framework aims to accelerate the development of evidence-based practices that optimize patient outcomes in this rapidly advancing field. As ESS continues to evolve toward addressing more complex pathologies, this living framework provides a practical, adaptable resource that can mature alongside technological innovations and expanding applications.

## Declaration of competing interest

The authors declare that they have no known competing financial interests or personal relationships that could have appeared to influence the work reported in this paper.

## Figures and Tables

**Table:** 

Domains	Subdomains	Working basis (n)	Add-ons (n)	Total number of items (n)	Proportion of add-ons (%)
Patient characteristics	Demographics	11	2	13	15.4
	Comorbidities	7	4	11	36.4
	Initial complaint	6	2	8	25
	Severity assessment	19	3	22	13.6
	Ongoing treatment(s)	11	0	11	0
Practice	Clinical	20	1	21	4.8
	Surgical	35	2	37	5.4
	Technical	27	1	28	3.6
Outcomes	Clinical	16	1	17	5.9
	Complications	13	2	15	13.3
**Total**		**165**	**18**	**183**	**9.8**

**Figure:**
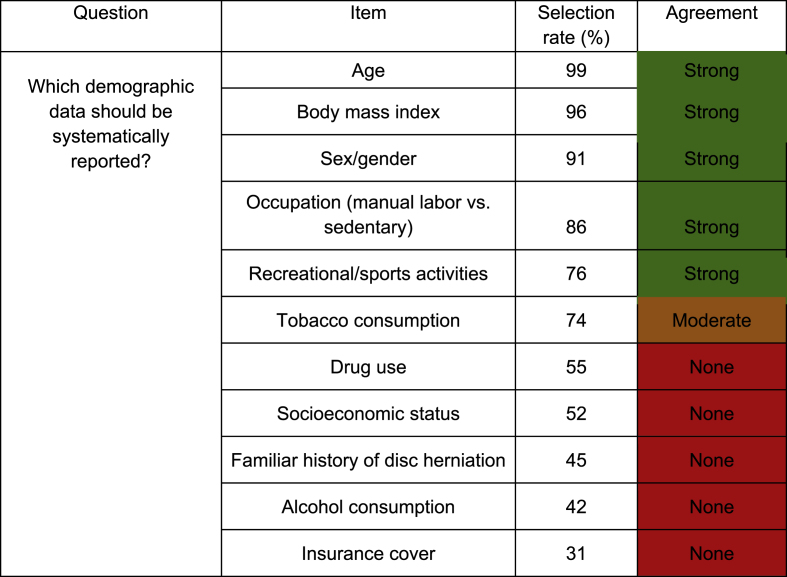

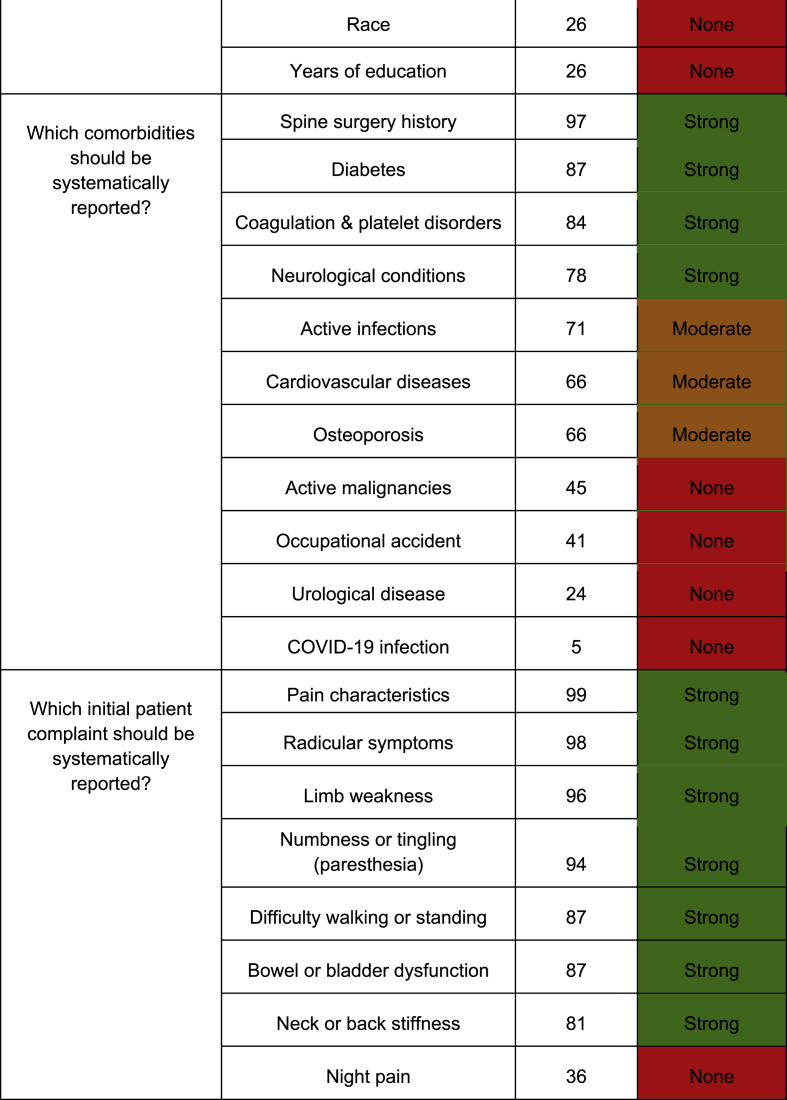

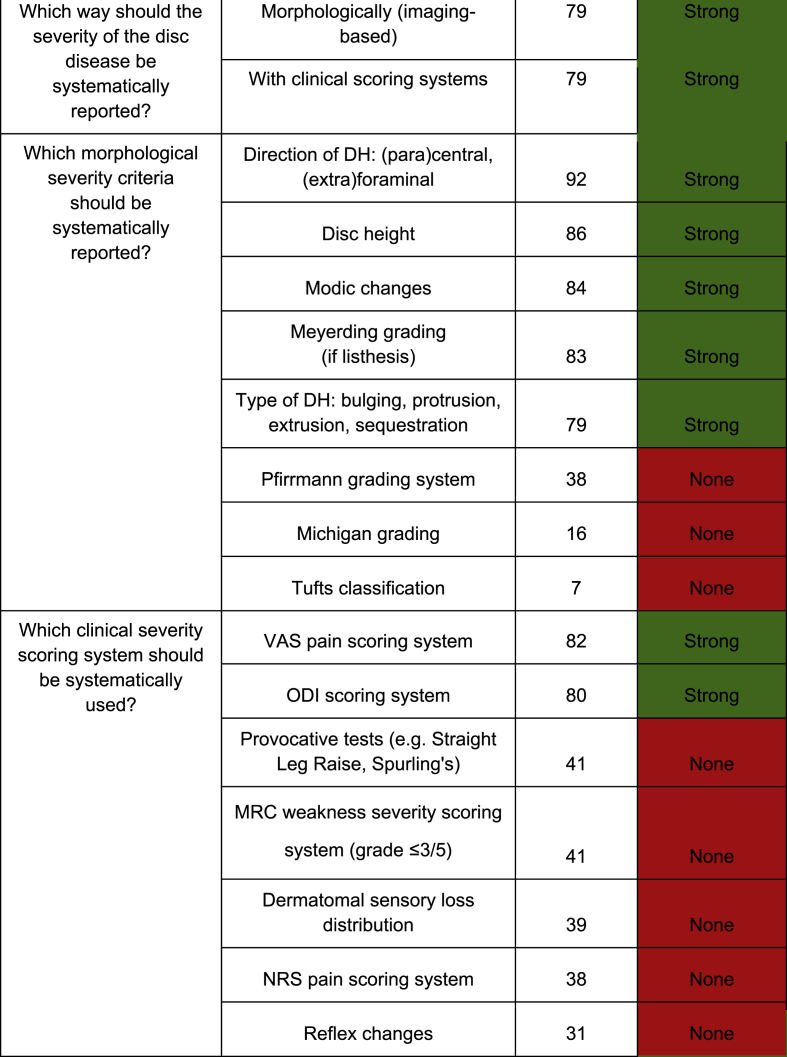

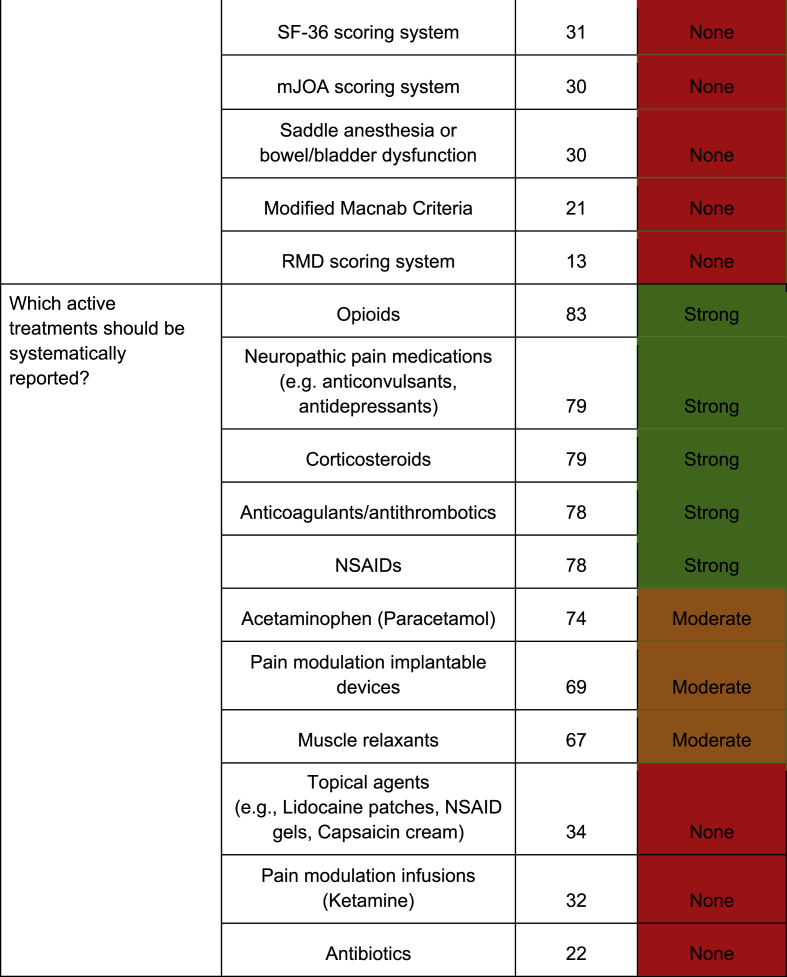
DH: disc herniation; MRC: Medical Research Council; mJOA: Modified Japanese Orthopaedic Association; NRS: Numeric Rating Scale; NSAIDs: Nonsteroidal Anti-Inflammatory Drugs; ODI: Oswestry Disability Index; RMD: Roland-Morris Disability; SF-36: Short Form Health Survey; VAS: Visual Analog Scale.

**Figure:**
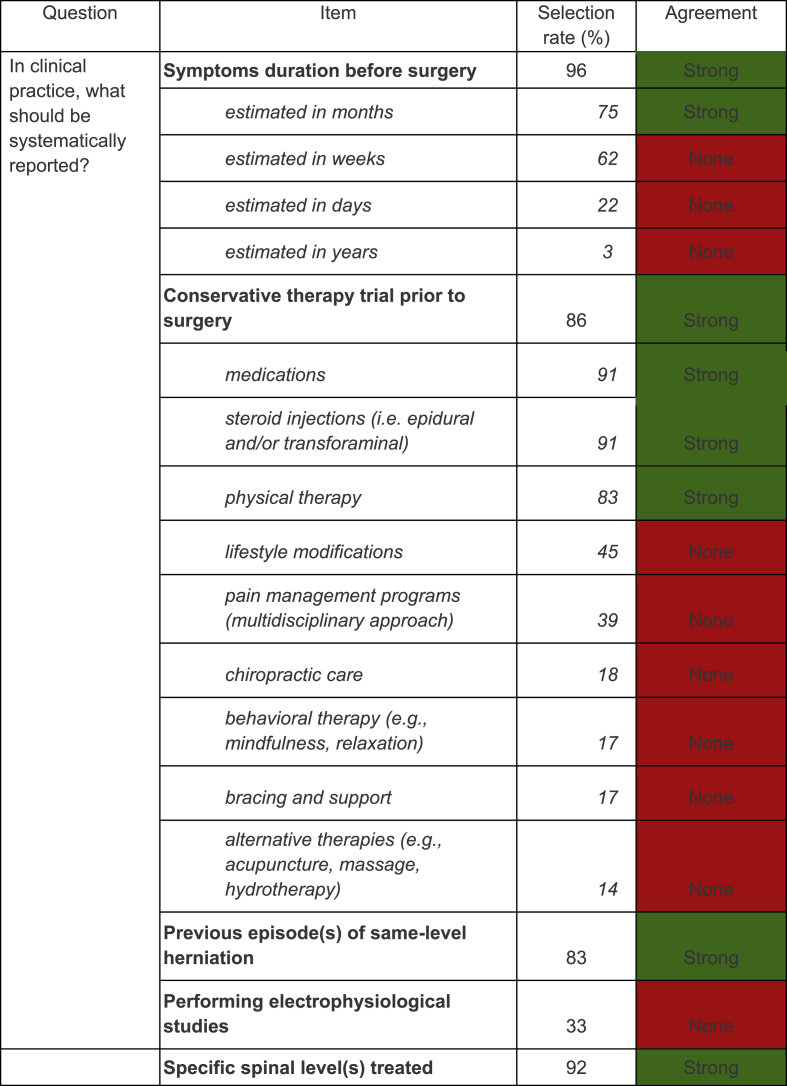

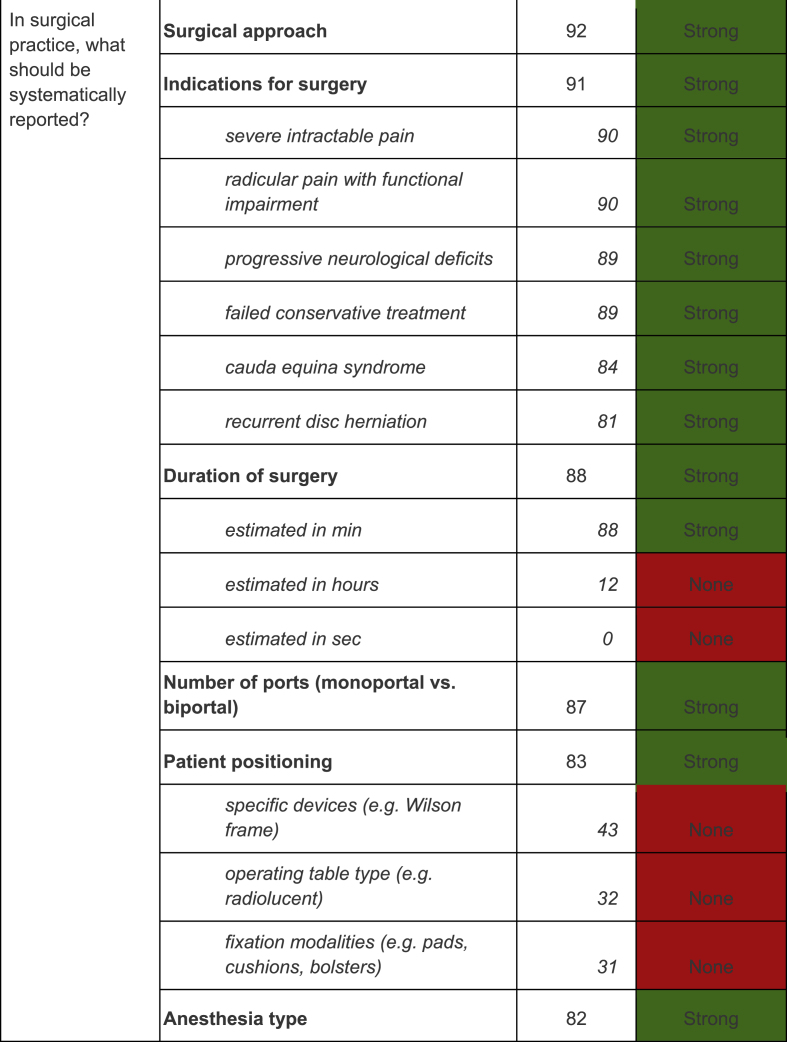

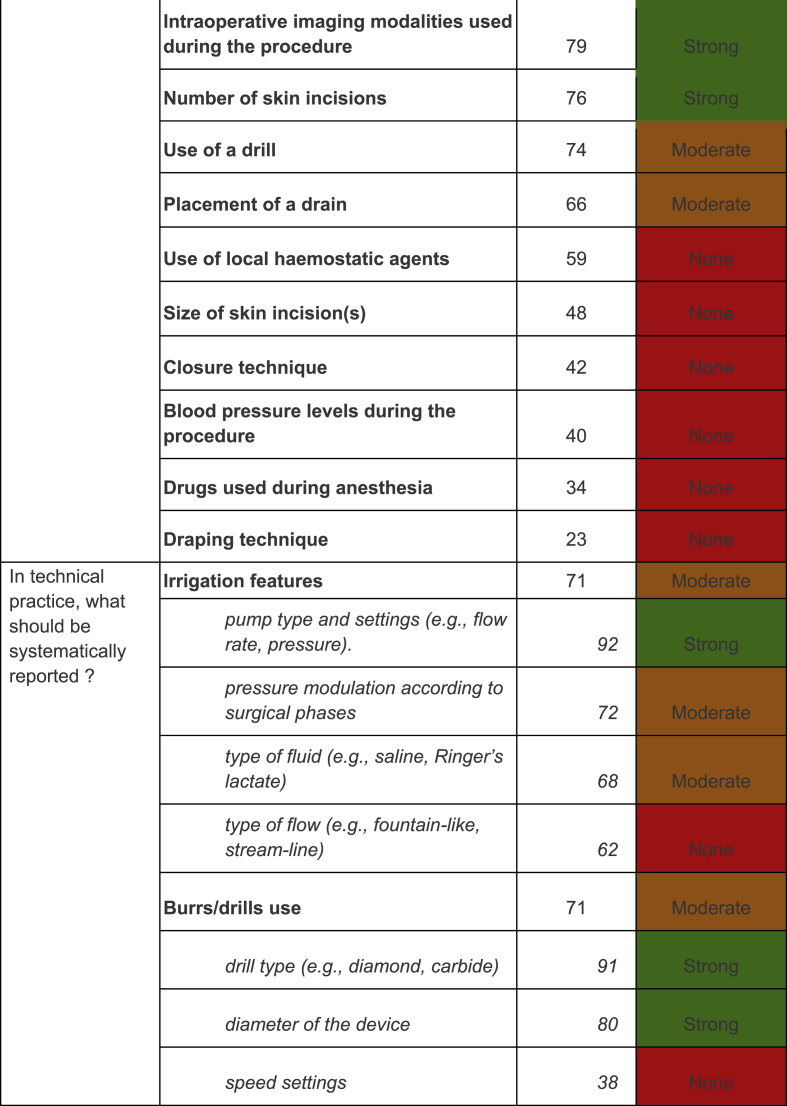

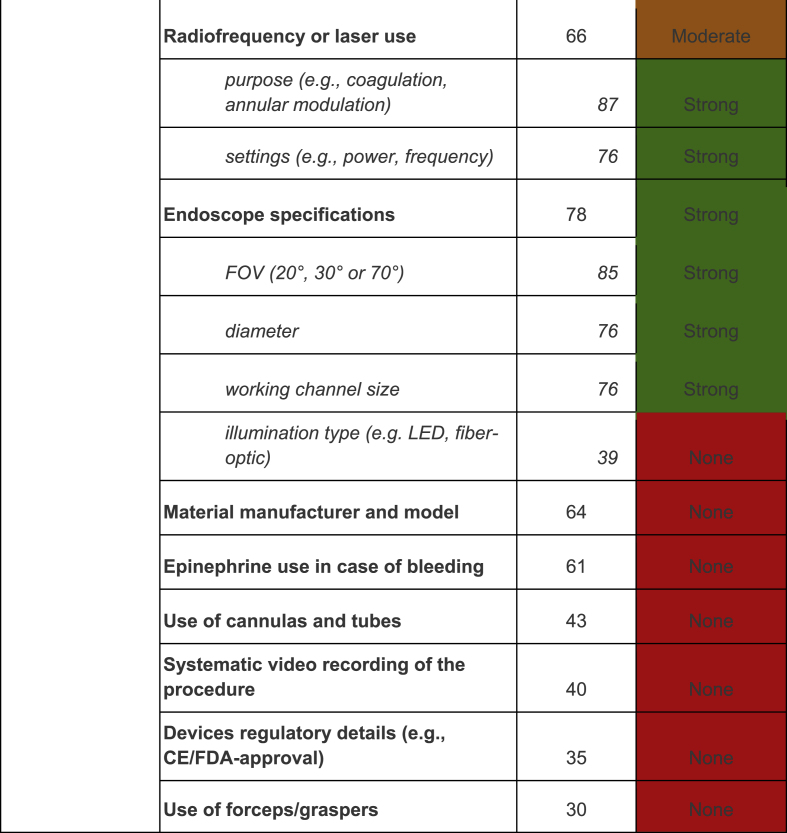
CE: Conformité Européene; FDA: Food and Drug Administration; FOV: field of view.

**Figure:**
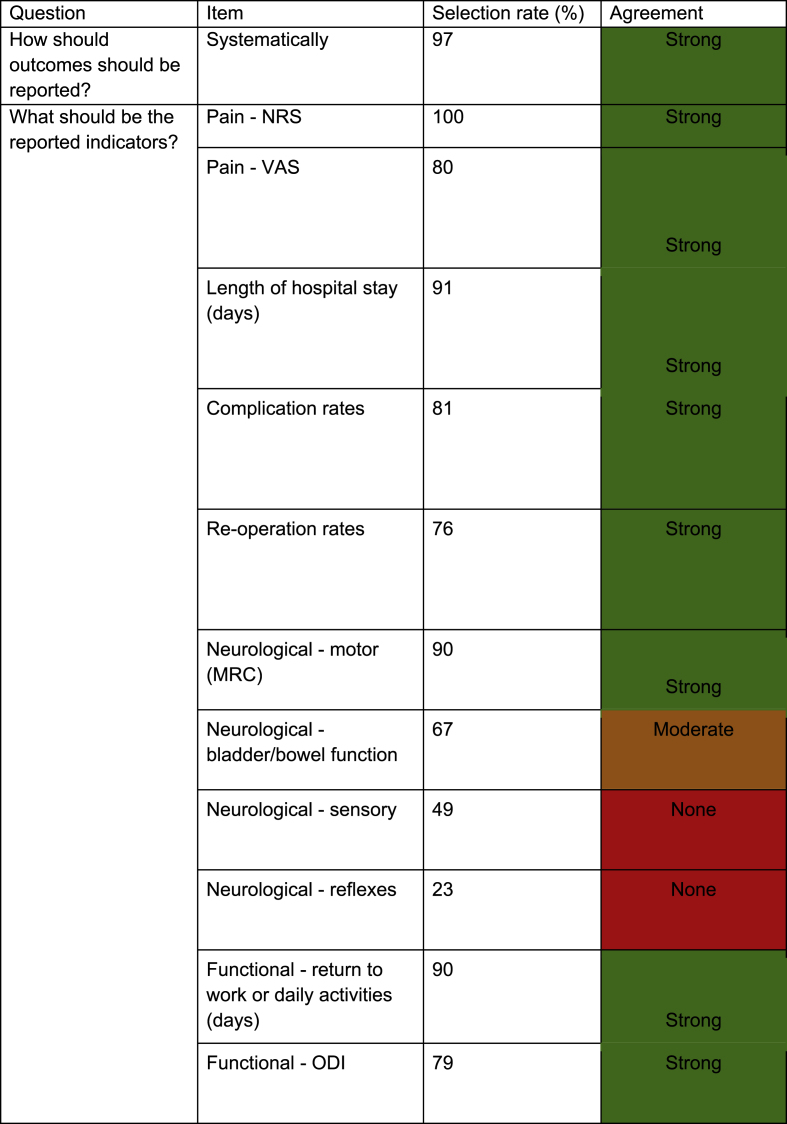

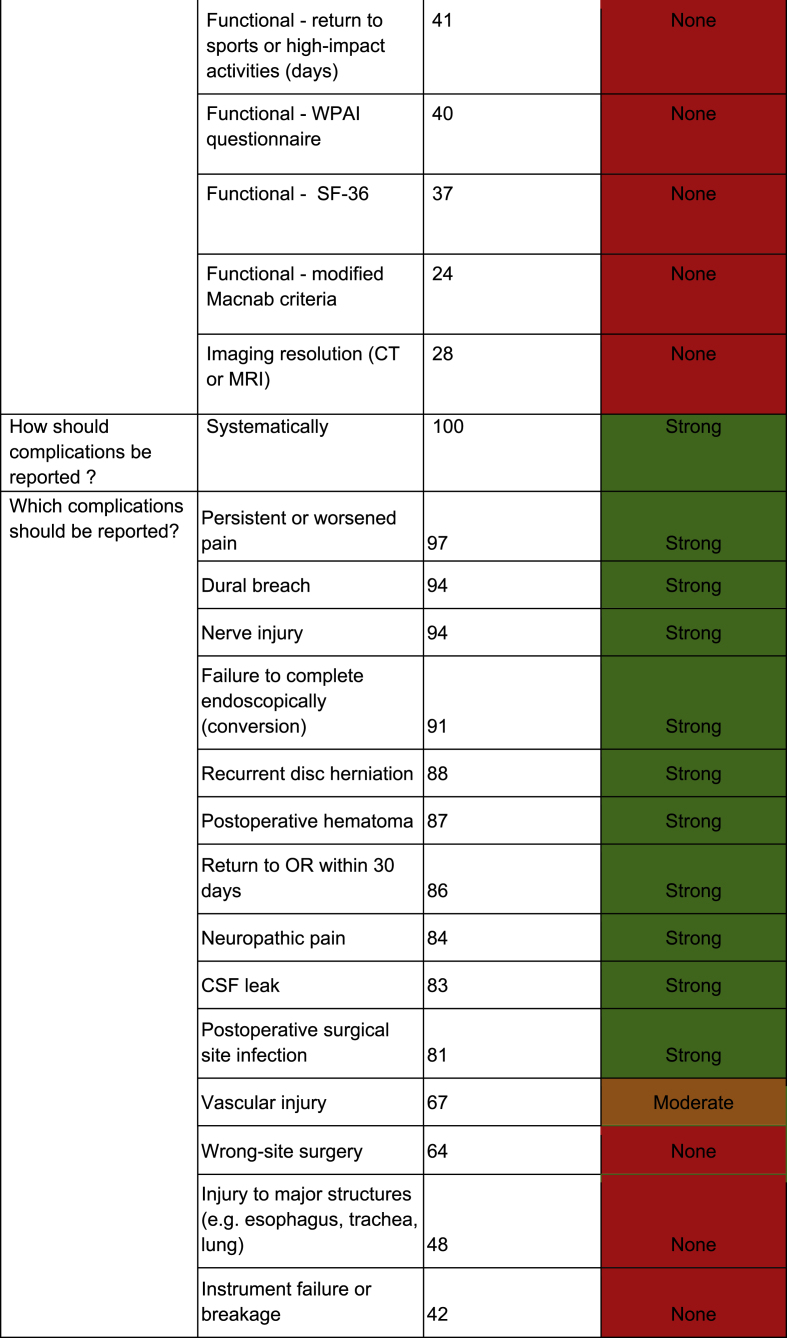
CSF: cerebrospinal fluid; MRC: Medical Research Council; NRS: Numeric Rating Scale; ODI: Oswestry Disability Index; OR: operating room; SF-36: Short Form Health Survey; VAS: Visual Analog Scale, WPAI: Work Productivity and Activity Impairment.
