# Supplementary material for: Spine endoscopic surgery establishment for disc disease (Neurocore-SENSED): an open and decentralized consensus
Source: Brain Spine. 2025 Sep 13;5:105604. doi: 10.1016/j.bas.2025.105604 (PMC12464703; doi:10.1016/j.bas.2025.105604)
Supplement: Multimedia component 1 [file mmc1.docx]

| Checklist description | Reporting criteria | Page # |
| --- | --- | --- |
| Patient characteristics | | |
| 1. Provide demographic information of the patients. | ☐ Age  ☐ Body mass index  ☐ Sex and/or gender  ☐ Occupation (manual labor vs sedentary)  ☐ Recreational/sports activities  ☐ Tobacco consumption |  |
| 1. List and describe all relevant comorbidities of the patients. | ☐ Spine surgery history  ☐ Diabetes  ☐ Coagulation & platelet disorders  ☐ Neurological conditions  ☐ Active infections  ☐ Cardiovascular diseases  ☐ Osteoporosis |  |
| 1. Describe initial patient complaints. | ☐ Pain characteristics  ☐ Radicular symptoms  ☐ Limb weakness  ☐ Numbness or tingling (paresthesia)  ☐ Difficulty walking or standing  ☐ Bowel or bladder dysfunction  ☐ Neck or back stiffness |  |
| 1. Describe the method of reporting the severity of the disc disease. | ☐ Morphologically (imaging-based)  ☐ Clinical scoring systems |  |
| 1. Describe the morphological (imaging-based) severity criteria for reporting the severity of the disc disease. | ☐ Direction of DH: (para)central, Extra (foraminal)  ☐ Disc height  ☐ Modic changes  ☐ Meyerding grading (if listhesis)  ☐ Type of DH: bulging, protrusion, extrusion, sequestration |  |
| 1. Describe clinical severity scoring systems used for reporting the severity of the disc disease. | ☐ VAS pain scoring system  ☐ ODI scoring system |  |

| Checklist description | Reporting criteria | Page # |
| --- | --- | --- |
| Patient characteristics | | |
| 1. Specify any active treatments and associated details (dosage, frequency). | ☐ Opioids  ☐ Neuropathic pain medications (e.g. anticonvulsants, antidepressants)  ☐ Corticosteroids  ☐ Anticoagulants/antithrombotics  ☐ NSAIDs  ☐ Acetaminophen (Paracetamol)  ☐ Pain modulation implantable devices  ☐ Muscle relaxants |  |

| Checklist description | Reporting criteria | Page # |
| --- | --- | --- |
| Practices | | |
| 1. Describe clinical practice features related to ESS. | ☐ Symptom duration before surgery (estimated in months)  ☐ Conservative therapy trial prior to surgery  ☐ Medications  ☐ Steroid injections (i.e. epidural and/or d transforaminal)  ☐ Physical therapy  ☐ Previous episode(s) of same-level herniation |  |
| 1. Describe surgical practice features related to ESS. | ☐ Specific spinal level(s) treated  ☐ Surgical approach  ☐ Indications for surgery  ☐ Severe intractable pain  ☐ Radicular pain with functional d impairment  ☐ Progressive neurological deficits  ☐ Failed conservative treatment  ☐ Cauda equina syndrome  ☐ Recurrent disc herniation  ☐ Duration of surgery (in minutes)  ☐ Number of ports (monoportal vs. biportal)  ☐ Anesthesia type  ☐ Intraoperative imaging modalities used during the procedure  ☐ Number of skin incisions  ☐ Use of a drill  ☐ Placement of a drain |  |
| 1. Describe technical details related to ESS. | ☐ Irrigation features  ☐ Pump type and settings (e.g., flow d rate, pressure)  ☐ Pressure modulation according to t surgical phases  ☐ Type of fluid (e.g., saline, Ringer’s d lactate)  ☐ Burrs/drills use  ☐ Drill type (e.g., diamond, carbide)  ☐ Diameter of the device  ☐ Radiofrequency or laser use  ☐ Purpose (e.g., coagulation, annular d modulation)  ☐ Settings (e.g., power, frequency)  ☐ Endoscopic specifications  ☐ FOV (*20°, 30° or 70°)*  ☐ Diameter  ☐ Working channel size |  |

| Checklist description | Reporting criteria | Page # |
| --- | --- | --- |
| Outcomes | | |
| 1. Describe notable outcomes indicators following ESS. | ☐ Pain - NRS  ☐ Pain - VAS  ☐ Length of hospital stay (days)  ☐ Complication rates  ☐ Re-operation rates  ☐ Neurological - motor (MRC)  ☐ Neurological - bladder/bowel function  ☐ Functional - return to work or daily activities (days)  ☐ Functional - ODI |  |
| 1. Report complications related to ESS. | ☐ Persistent or worsened pain  ☐ Dural breach  ☐ Nerve injury  ☐ Failure to complete endoscopically (conversion)  ☐ Recurrent disc herniation  ☐ Post-operative hematoma  ☐ Return to OR within 30 days  ☐ Neuropathic pain  ☐ CSF leak  ☐ Postoperative surgical site infection  ☐ Vascular injury |  |

***Abbreviations:*** *DH: disc herniation; VAS: Visual Analog Scale; ODI: Oswestry Disability Index; NSAIDs: Nonsteroidal Anti-Inflammatory Drugs; ESS: Endoscopic Spine Surgery; FOV: field of view. NRS: Numeric Rating Scale; MRC: Medical Research Council; OR: operating room; CSF: cerebrospinal fluid.*
