# Supplementary material for: Spine endoscopic surgery establishment for disc disease (Neurocore-SENSED): an open and decentralized consensus
Source: Brain Spine. 2025 Sep 13;5:105604. doi: 10.1016/j.bas.2025.105604 (PMC12464703; doi:10.1016/j.bas.2025.105604)

Spine Endoscopic Surgery for Disc Disease (**SENSE-D**): consensus-based guidelines

Round 1: comprehensive report

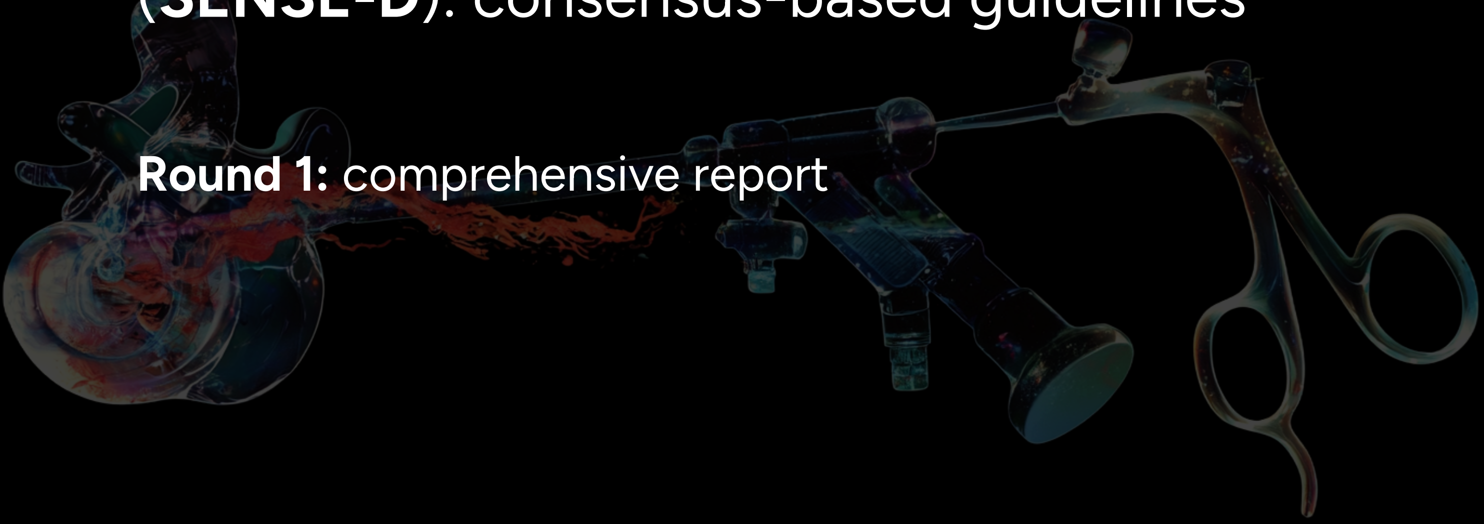

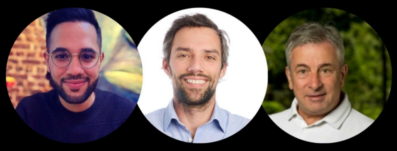

Mejdeddine Al Barajraji, MD
Richard Assaker, MD, PhD
Thibault Remacle, MD, PhD

Sciense
mejdi@sciense.org
447 Broadway, 2nd Floor
New York, NY 10013

neurocore

This open report details results and additional propositions from each round, ensuring transparency and informing subsequent rounds.

Inclusion: endorsed by ≥75% of participants are immediately added to the core outcome set (COS) ✅
Exclusion: with <50% endorsement are removed—a call can be made by any participant ❌
Reevaluation: with more than 50 and less than 75% endorsement are reconsidered in the next round 🔄

Note that propositions

- are submitted for consideration a maximum of two rounds.
- not meeting COS criteria may still inform fork projects of dedicated working groups.

Concerning our treatment of additional propositions or insights, 'ADD-ONS,' they can be either:

- directly added to the next round ➕
- covered or integrated with others ⬇️
- considered valuable insights, not forming direct propositions 💡

Sciense
mejdi@sciense.org
447 Broadway, 2nd Floor
New York, NY 10013

Global participation

Region	Count
Europe	47
Asia	11
North America	10
Africa	6
South America	3

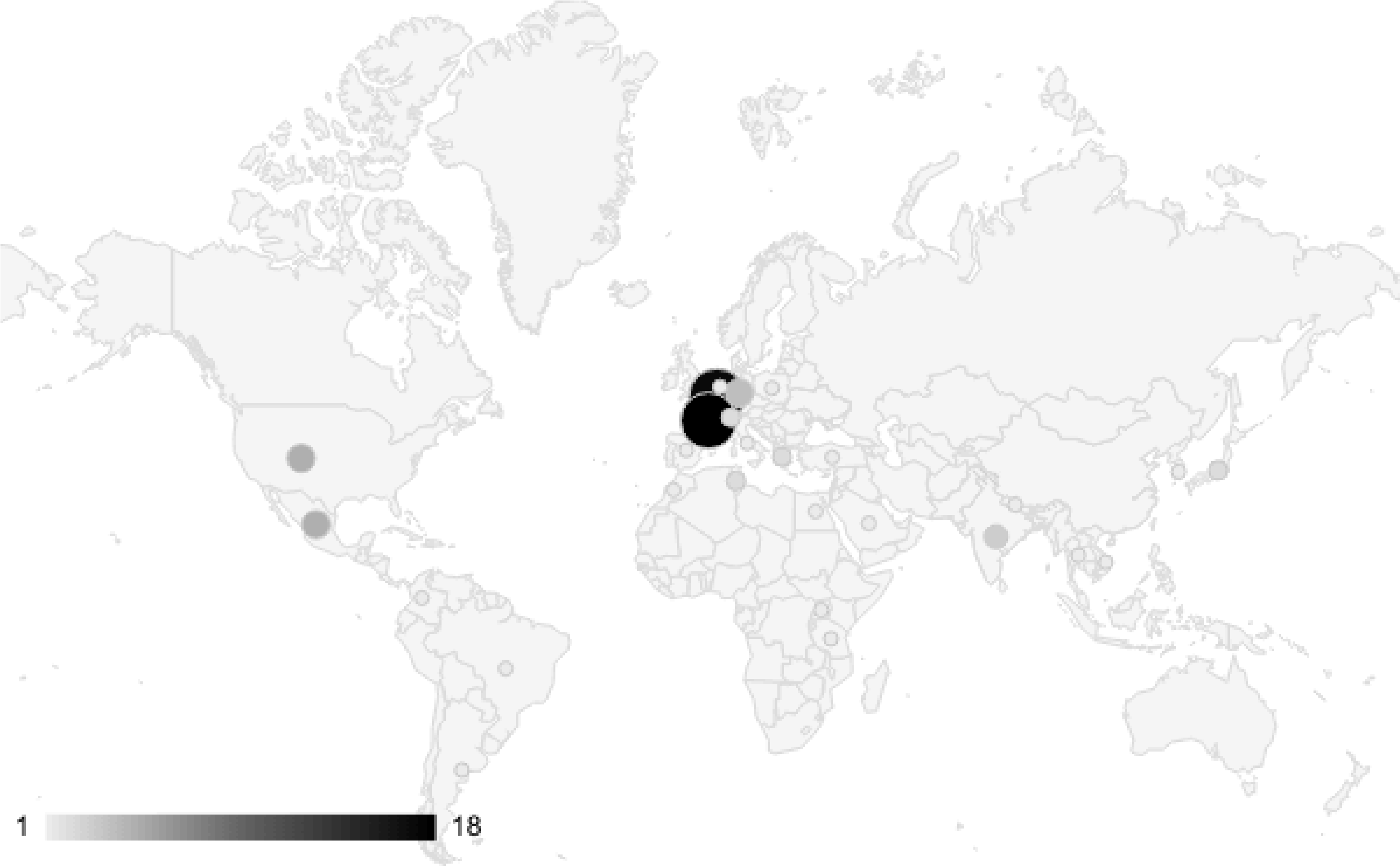

neurocore

Participating experts

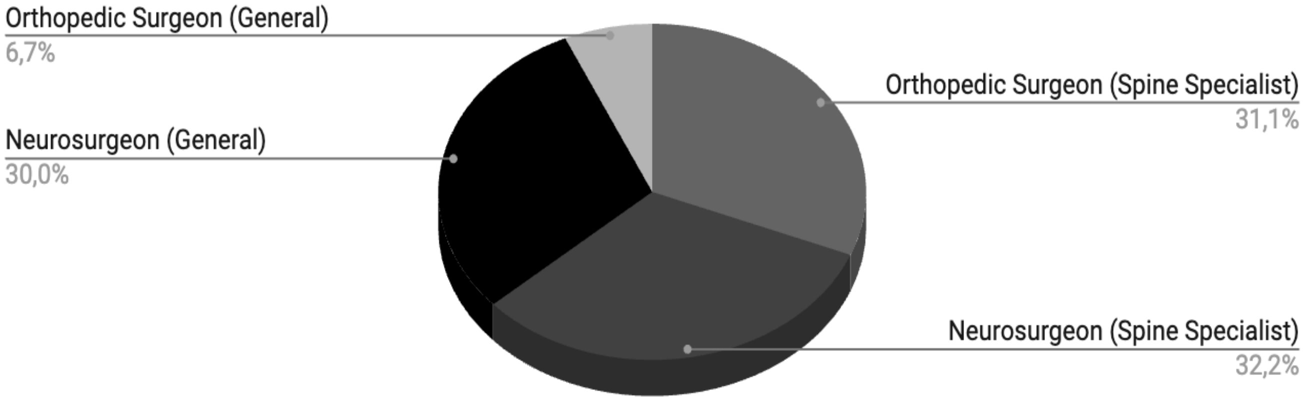

Years of Surgical Practice (n=77)

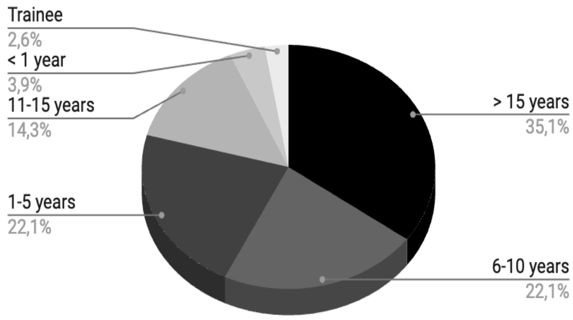

Herniated discs treated annually (n=77)

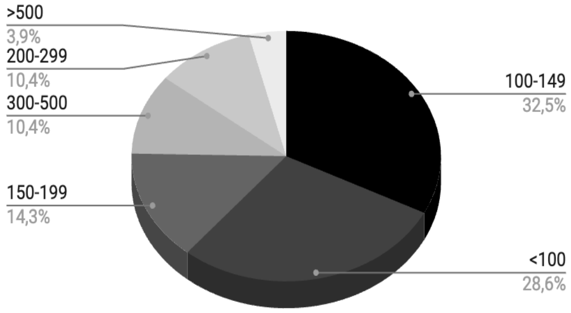

Sciense
mejdi@sciense.org
447 Broadway, 2nd Floor
New York, NY 10013

neurocore

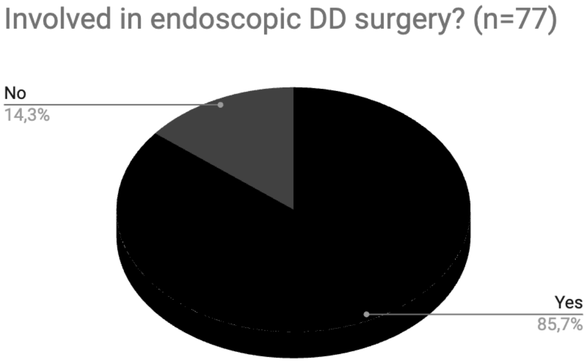

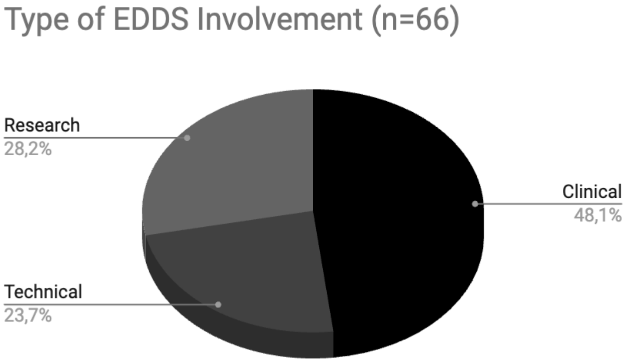

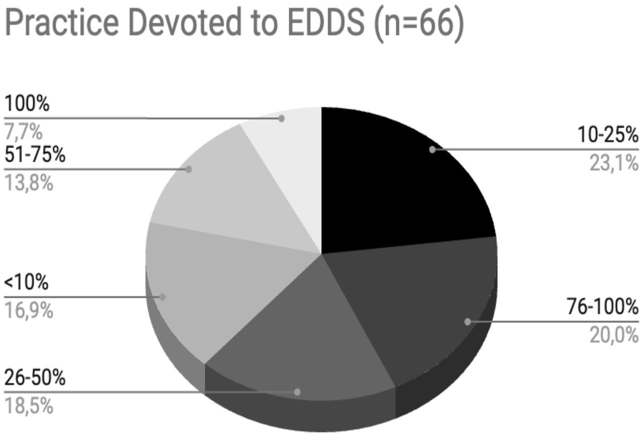

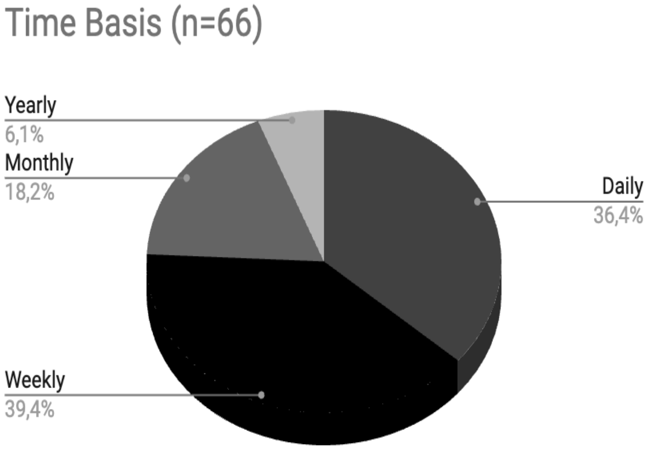

Sciense
mejdi@sciense.org
447 Broadway, 2nd Floor
New York, NY 10013

neurocore

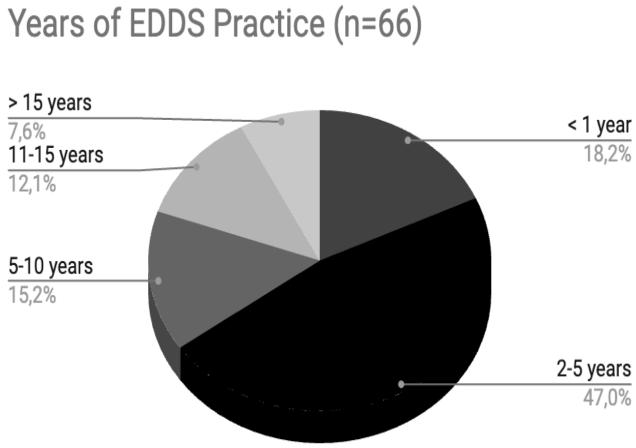

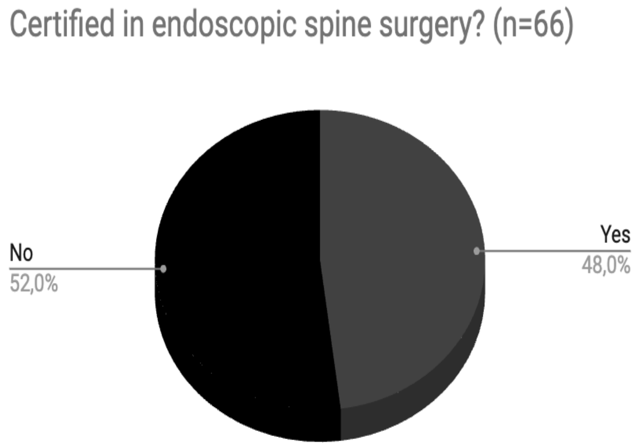

Sciense
mejdi@sciense.org
447 Broadway, 2nd Floor
New York, NY 10013

neurocore

Baseline

Which patient variables should be systematically reported in studies mentioning EDDS?

Demographics:

77 responses

age	99% ✅	76 responses
sex/gender	91% ✅	70 responses
body mass index (BMI)	96% ✅	74 responses
occupation (manual labor vs. sedentary)	86% ✅	66 responses
drug use	55% 🔄	42 responses
tobacco consumption	61% 🔄	47 responses
alcohol consumption	42% ❌	32 responses
insurance cover	31% ❌	24 responses
socioeconomic status	51% 🔄	39 responses
race	26% ❌	20 responses
years of education	26% ❌	20 responses
no opinion	1%	1 response

ADD-ONS

- Familiar history of disc herniation
- Recreational/ sports activities

Comorbidities:

77 responses

coagulation & platelet disorders	84% ✅	65 responses
diabetes	87% ✅	67 responses
spine surgery history	97% ✅	75 responses
cardiovascular diseases	66% 🔄	51 responses
neurological conditions	78% ✅	60 responses
active malignancies	45% ❌	35 responses
active infections	58% 🔄	45 responses
no opinion	0%	No responses

ADD-ONS

- Urological disease
- Occupational accident
- COVID infection
- Osteoporosis
- Obesity

Sciense
mejdi@sciense.org
447 Broadway, 2nd Floor
New York, NY 10013

neurocore

Initial complaint:

77 responses

Pain Characteristics	99%	✅	76 responses
Limb Weakness	96%	✅	74 responses
Numbness or Tingling (Paresthesia)	94%	✅	72 responses
Neck or Back Stiffness	70%	🔄	54 responses
Difficulty Walking or Standing	87%	✅	67 responses
Bowel or Bladder Dysfunction	87%	✅	67 responses
no opinion	0%		No responses

ADD-ONS

- Night pain

Severity assessment:

77 responses

Morphological (imaging-based)	79%	✅	61 responses
Clinical Severity Scoring Systems	79%	✅	61 responses

Sciense
mejdi@sciense.org
447 Broadway, 2nd Floor
New York, NY 10013

neurocore

Which Morphological Severity Assessment ?

61 responses

Option	%		Responses
Direction of DH: (para)central, (extra)foraminal	92%	✅	56 responses
Type of DH: bulging, protrusion, extrusion, sequestration	79%	✅	48 responses
Pfirrmann grading system	38%	❌	23 responses
Tufts Classification	7%	❌	4 responses
Michigan Grading	16%	❌	10 responses

ADD-ONS

- Disc height
- Meyerding grading (if listhesis)

Which Clinical Severity Scoring Systems?

61 responses

Option	%		Responses
Pain: Visual Analog Scale (VAS)	82%	✅	50 responses
Pain: Numeric Rating Scale (NRS)	38%	❌	23 responses
Sensory loss (dermatomal distribution)	39%	❌	24 responses
Reflex changes	31%	❌	19 responses
Disability & functional impairment : Modified Macnab Criteria	21%	❌	13 responses
Disability & functional impairment: Oswestry Disability Index (ODI)	80%	✅	49 responses
Disability & functional impairment: Roland-Morris Disability Questionnaire (RMDQ)	13%	❌	8 responses
Disability & functional impairment: SF-36 (Short Form Health Survey)	31%	❌	19 responses
Provocative Tests (e.g. Straight Leg Raise, Spurling's)	41%	❌	25 responses
Modified Japanese Orthopaedic Association (mJOA) Scoring System	30%	❌	18 responses
Saddle anesthesia or bowel/bladder dysfunction	30%	❌	18 responses
Severe weakness (MRC grade ≤3/5)	41%	❌	25 responses

Sciense
mejdi@sciense.org
447 Broadway, 2nd Floor
New York, NY 10013

neurocore

Ongoing treatment(s):

77 responses

Treatment	%		Responses
Anticoagulants/antithrombotics	78%	✅	60 responses
Nonsteroidal Anti-Inflammatory Drugs (NSAIDs)	78%	✅	60 responses
Acetaminophen (Paracetamol)	53%	🔄	41 responses
Opioids	83%	✅	64 responses
Muscle Relaxants	51%	🔄	39 responses
Neuropathic Pain Medications (e.g. anticonvulsants, antidepressants)	79%	✅	61 responses
Topical Agents (e.g. Lidocaine patches, NSAID gels, Capsaicin cream)	34%	❌	26 responses
Corticosteroids	79%	✅	61 responses
Pain Modulation: implantable devices	56%	🔄	43 responses
Pain Modulation: Ketamine infusions	32%	❌	25 responses
Antibiotics	22%	❌	17 responses
no opinion	0%		No responses

Sciense
mejdi@sciense.org
447 Broadway, 2nd Floor
New York, NY 10013

neurocore

Practice items

Which practices should be systematically defined in studies reporting EDDS?

Clinical aspects:

77 responses

Duration of symptoms prior to surgery	96% ✅	74 responses

69 responses

A days	22% ❌	15 responses
B months	75% ✅	52 responses
C years	3% ❌	2 responses

ADD-ONS

- Weeks

Previous episode(s) of same-level herniation	83% ✅	64 responses

Trial of conservative therapy prior to surgery	86% ✅	66 responses

Sciense
mejdi@sciense.org
447 Broadway, 2nd Floor
New York, NY 10013

neurocore

Lifestyle modifications	45% ✗	30 responses
Medications	73% 🔄	48 responses
Physical Therapy	83% ✓	55 responses
Steroid Injections (i.e. epidural and/or transforaminal)	91% ✓	60 responses
Pain Management Programs (Multidisciplinary Approach)	39% ✗	26 responses
Behavioral Therapy (e.g. Mindfulness, Relaxation)	17% ✗	11 responses
Bracing and Support	17% ✗	11 responses
Chiropractic Care	18% ✗	12 responses
Alternative Therapies (e.g. acupuncture, massage, hydrotherapy)	14% ✗	9 responses

Electrophysiological Studies	35% ✗	27 responses
↳ EMG	93%	26 responses
↳ Nerve conduction studies	46%	13 responses
↳ Dermatomal Somatosensory Evoked Potentials (dSSEPs)	32%	9 responses
↳ Motor Evoked Potentials (MEPs)	29%	8 responses
I have no opinion regarding clinical practices reporting	1%	1 response

Sciense
mejdi@sciense.org
447 Broadway, 2nd Floor
New York, NY 10013

neurocore

Surgical aspects:

77 responses

Item	%	Status	Responses
Specific spinal level(s) treated	92%	✅	71 responses
Indication for surgery	91%	✅	70 responses
↳ Cauda Equina Syndrome	84%	✅	59 responses
↳ Progressive Neurological Deficits	89%	✅	62 responses
↳ Severe, Intractable Pain	90%	✅	63 responses
↳ Radicular Pain with Functional Impairment	90%	✅	63 responses
↳ Failed Conservative Treatment	89%	✅	62 responses
↳ Recurrent Disc Herniation	81%	✅	57 responses

Item	%	Status	Responses
Patient positioning	66%	🔄	51 responses
Patient fixation details (e.g. pads, cushions, bolsters)	31%	❌	24 responses
Operating table type (e.g., radiolucent)	32%	❌	25 responses
Specific positioning devices (e.g., Wilson frame, pillow supports).	43%	❌	33 responses

Sciense
mejdi@sciense.org
447 Broadway, 2nd Floor
New York, NY 10013

neurocore

Item	%	Status	Responses
Draping technique	23%	❌	18 responses
Surgical Approach	92%	✅	71 responses
Anesthesia Type	82%	✅	63 responses
Number of skin incisions	69%	🔄	53 responses
Number of ports (monoportal vs. biportal)	87%	✅	67 responses
Size of skin incision(s)	51%	🔄	39 responses
↳ A mm	87%	✅	33 responses
↳ B cm	13%	❌	5 responses
Duration of Surgery	88%	✅	68 responses
↳ A sec	0%	❌	No responses
↳ B min	88%	✅	57 responses
↳ C hours	12%	❌	8 responses
Intraoperative imaging modalities used during the procedure	68%	🔄	52 responses
Use of a drill	69%	🔄	53 responses
Closure technique	42%	❌	32 responses
Placement of a drain	68%	🔄	52 responses
Use of local haemostatic agents	60%	🔄	46 responses

Sciense
mejdi@sciense.org
447 Broadway, 2nd Floor
New York, NY 10013

Bone Wax	65% 🔄	30 responses
Absorbable Gelatin Sponges (e.g., Gelfoam)	72% 🔄	33 responses
Gelatin Matrix (e.g., Floseal)	85% ✅	39 responses
Oxidized regenerated cellulose (e.g., Surgicel)	65% 🔄	30 responses
Microfibrillar collagen (e.g., Avitene)	43% ❌	20 responses
Fibrin sealants (e.g., TachoSil)	76% ✅	35 responses
I have no opinion regarding surgical practices reporting	3%	2 responses

- Drugs used during anesthesia
- Blood pressure levels during the procedure

Technical aspects:

77 responses

Material Manufacturer and Model	66% 🔄	51 responses
Devices regulatory details (e.g., CE/FDA-approval)	35% ❌	27 responses
Endoscope Specifications	66% 🔄	51 responses

Sciense
mejdi@sciense.org
447 Broadway, 2nd Floor
New York, NY 10013

neurocore

Item	Percentage	Status	Responses
Diameter	86%	✅	44 responses
Working channel size	82%	✅	42 responses
Field of view (e.g., 20°, 30°, or 70°)	88%	✅	45 responses
Illumination type (e.g., fiber-optic, LED)	45%	❌	23 responses

Item	Percentage	Status	Responses
Instrumentation Used: Cannulas and Tubes	43%	❌	33 responses
Type and size	88%	✅	30 responses
Outer and inner diameter	65%	🔄	22 responses
Length of the cannula	68%	🔄	23 responses

- Use of dilatators

Item	Percentage	Status	Responses
Instrumentation Used: Forceps/Graspers	30%	❌	23 responses
Type and size (e.g., rongeur, pituitary, or serrated)	92%	✅	23 responses
Specific purpose (e.g., herniated fragment removal, bone removal)	80%	✅	20 responses

Item	Percentage	Status	Responses
Instrumentation Used: Burrs/Drills	58%	🔄	45 responses
Drill type (e.g., diamond, carbide)	91%	✅	41 responses
Speed settings	64%	🔄	29 responses
Diameter of the drill or burr	80%	✅	36 responses

Item	Percentage	Status	Responses
Instrumentation Used: Radiofrequency or Laser Devices	53%	🔄	41 responses

Sciense
mejdi@sciense.org
447 Broadway, 2nd Floor
New York, NY 10013

neurocore

Settings used (e.g., power, frequency).	88% ✅	37 responses
Purpose (e.g., coagulation, annular modulation).	83% ✅	35 responses

Irrigation System	69% 🔄	53 responses
Irrigation pump type and settings (e.g., flow rate, pressure).	92% ✅	49 responses
Type of irrigation fluid (e.g., saline, Ringer's lactate).	70% 🔄	37 responses
Type of flow (e.g. fountain-like, stream-line)	70% 🔄	37 responses
Epinephrine use in case of bleeding	64% 🔄	34 responses

ADD-ONS

- Pressure modulation according to surgical phases

Systematic video recording of the procedure	40% ❌	31 responses
I have no opinion regarding clinical practices reporting	10%	8 responses

Sciense
mejdi@sciense.org
447 Broadway, 2nd Floor
New York, NY 10013

neurocore

Outcome items

Should we recommend reporting outcomes in a standardized way?

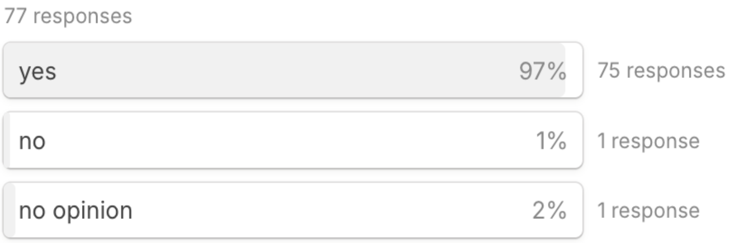

Sciense
mejdi@sciense.org
447 Broadway, 2nd Floor
New York, NY 10013

neurocore

Which way(s)?

75 responses

Option	Percentage	Status	Responses
Pain Reduction: VAS	80%	✅	60 responses
Pain Reduction: NRS	53%	🔄	40 responses
Neurological Improvement: bladder/bowel function	59%	🔄	44 responses
Neurological Improvement: Motor (MRC)	71%	🔄	53 responses
Neurological Improvement: Sensory Function	49%	❌	37 responses
Neurological Improvement: Reflexes	23%	🔄	17 responses
Functional Improvement: ODI	79%	✅	59 responses
Functional Improvement: Modified Macnab Criteria	24%	🔄	18 responses
Functional Improvement: Return to Work or Daily Activities (days)	61%	🔄	46 responses
Functional Improvement: Return to Sports or High-Impact Activities (days)	41%	❌	31 responses
Imaging resolution (CT or MRI)	28%	❌	21 responses
Patient-Reported: SF-36	37%	❌	28 responses
Complication Rates	81%	✅	61 responses
Reoperation Rates	76%	✅	57 responses
Length of Hospital Stay	63%	🔄	47 responses

ADD-ONS

- Work Productivity and Activity Impairment (WPAI) Questionnaire
- Contributions to labor productivity
- Return to work/sports

Sciense
mejdi@sciense.org
447 Broadway, 2nd Floor
New York, NY 10013

Should we recommend reporting complications in a standardized way?

77 responses

yes	100%	77 responses
no	0%	No responses
no opinion	0%	No responses

Sciense
mejdi@sciense.org
447 Broadway, 2nd Floor
New York, NY 10013

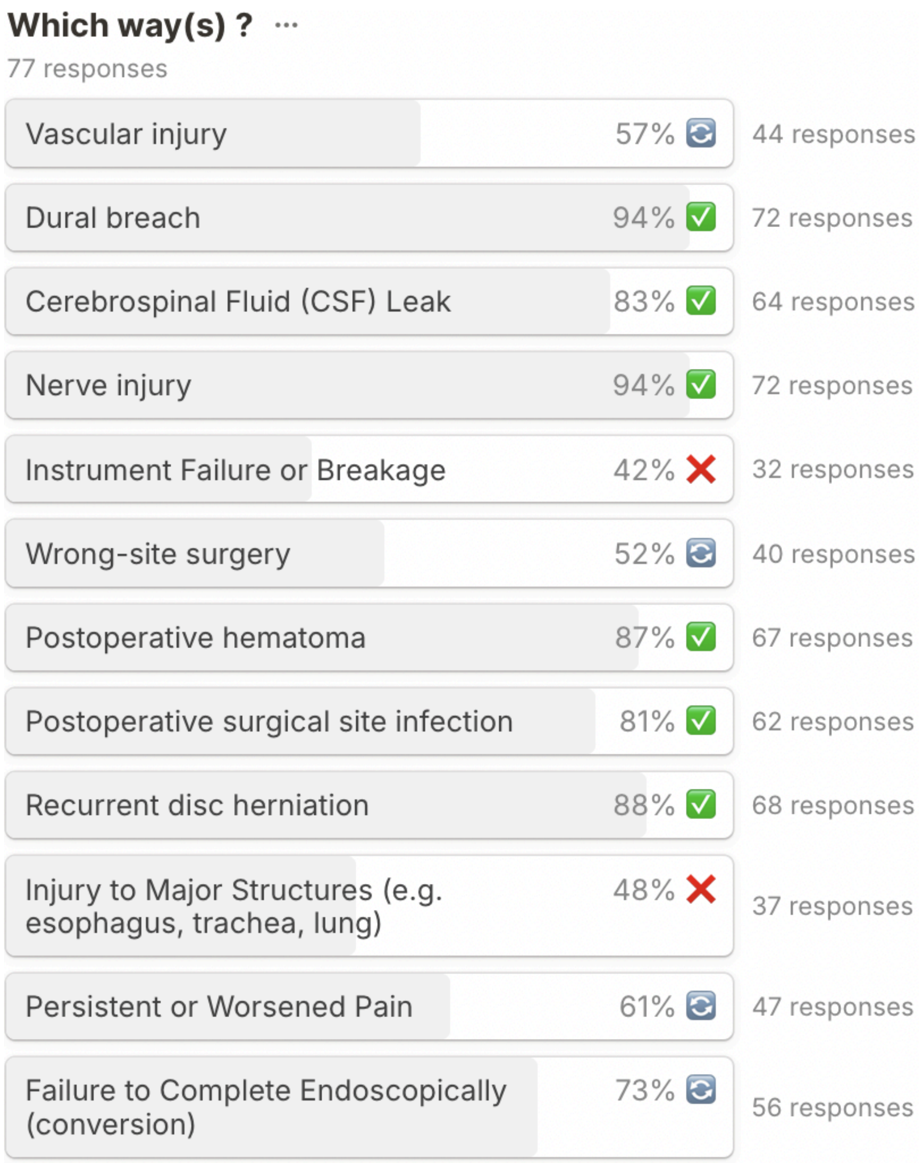

ADD-ONS

- Neuropathic pain
- Return to OR within 30 days

Sciense
mejdi@sciense.org
447 Broadway, 2nd Floor
New York, NY 10013

Perspectives

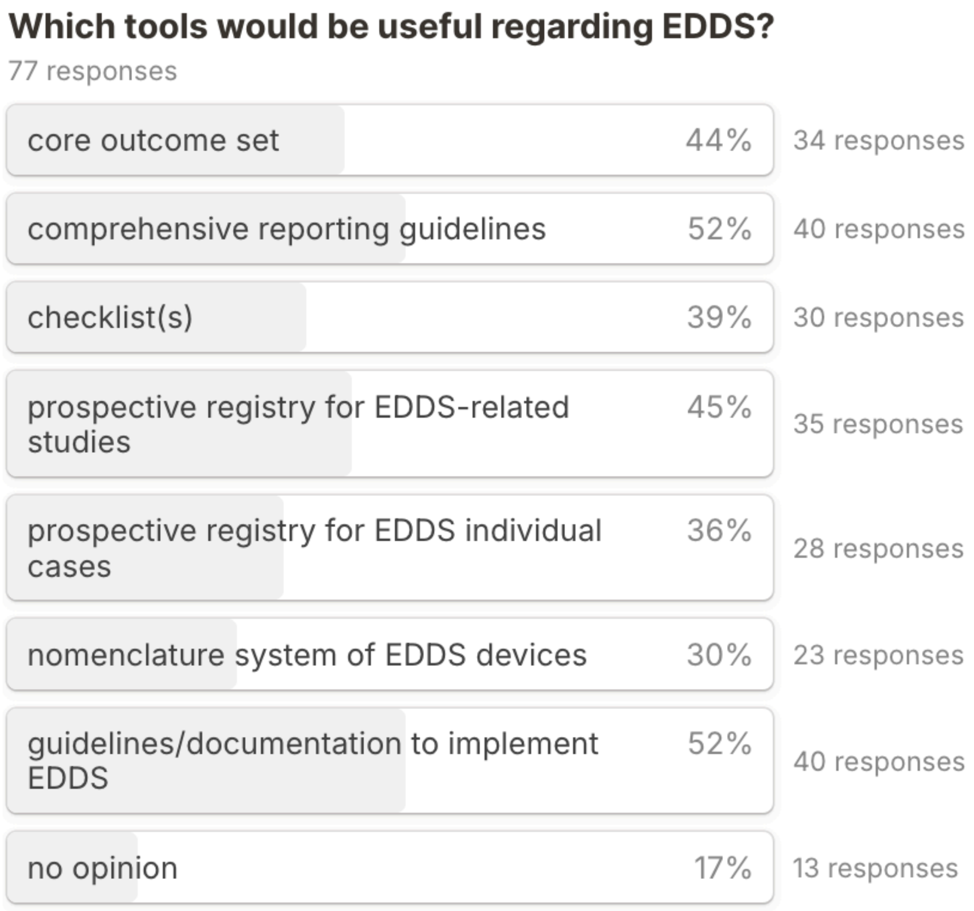

Sciense
mejdi@sciense.org
447 Broadway, 2nd Floor
New York, NY 10013

neurocore

Spine Endoscopic Surgery for Disc Disease (**SENSE-D**): consensus-based guidelines

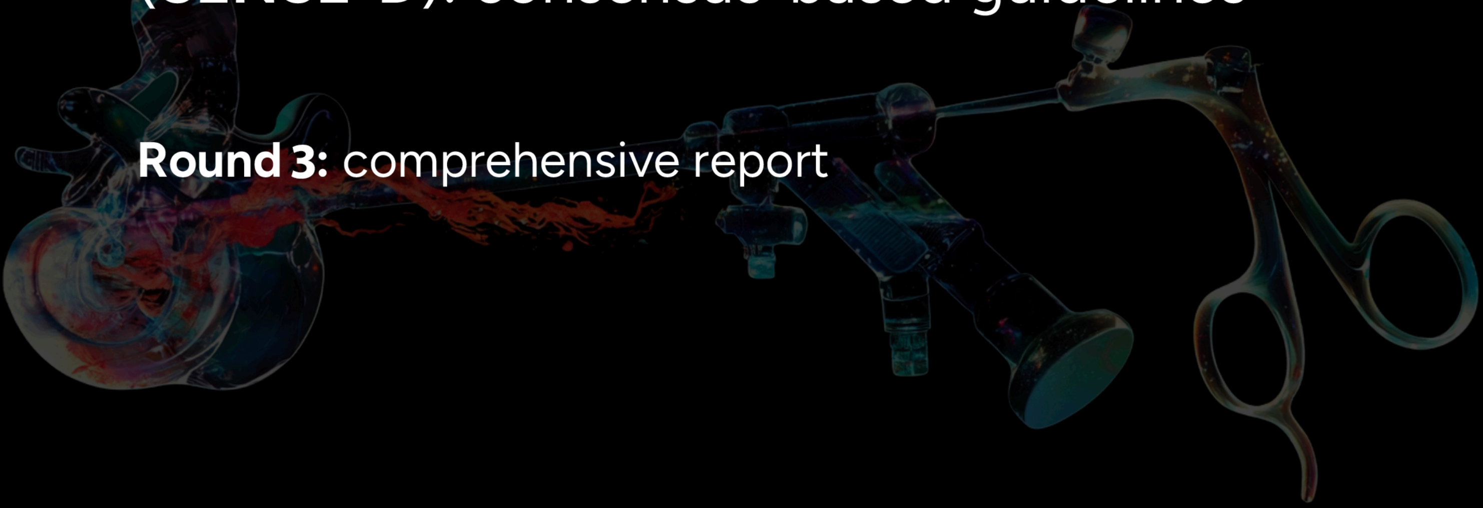

Round 3: comprehensive report

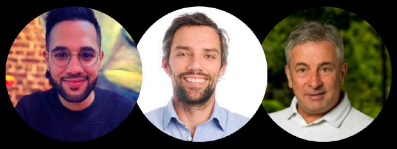

Mejdeddine Al Barajraji, MD
Richard Assaker, MD, PhD
Thibault Remacle, MD, PhD

Sciense
mejdi@sciense.org
447 Broadway, 2nd Floor
New York, NY 10013

neurocore

This open report details results and additional propositions from each round, ensuring transparency and informing subsequent rounds.

Inclusion: endorsed by ≥75% of participants are immediately added to the core outcome set (COS) ✅

Exclusion: with <50% endorsement are removed—a call can be made by any participant ❌

Reevaluation: with more than 50 and less than 75% endorsement are reconsidered in the next round 🔄

Note that propositions

- are submitted for consideration a maximum of two rounds.
- not meeting COS criteria may still inform fork projects of dedicated working groups.

Concerning our treatment of additional propositions or insights, 'ADD-ONS,' they can be either:

- directly added to the next round ➕
- covered or integrated with others ⬇️
- considered valuable insights, not forming direct propositions 💡

Sciense
mejdi@sciense.org
447 Broadway, 2nd Floor
New York, NY 10013

neurocore

I. Baseline items

Which patient variables should be systematically reported in studies mentioning EDDS?

Demographics:

58 responses

recreational/sports activities	76% ✅	44 responses
no opinion	24%	14 responses

Comorbidities:

58 responses

osteoporosis	66% ✅	38 responses
no opinion	34%	20 responses

Initial complaint:

58 responses

+ radicular symptoms	98% ✅	57 responses
night pain	36% ❌	21 responses
no opinion	2%	1 response

Severity assessment:

77 responses

Morphological (imaging-based)	79% ✅	61 responses
Clinical Severity Scoring Systems	79% ✅	61 responses

↓

58 responses

+ Modic changes	84% ✅	49 responses
no opinion	16%	9 responses

Sciense
mejdi@sciense.org
447 Broadway, 2nd Floor
New York, NY 10013

neurocore

II. Practice items

Which practices should be systematically defined in studies reporting EDDS?

<u>Clinical aspects:</u>

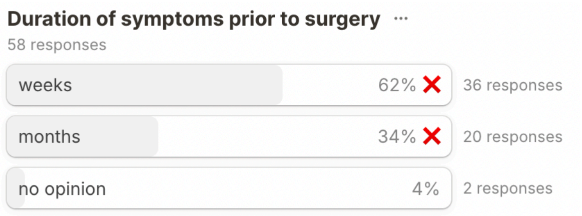

N.B : « months » already included in round 1, comparative reevaluation of "weeks" sub-item

<u>Technical aspects:</u>

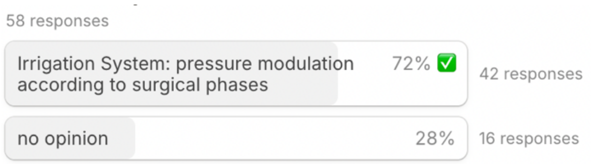

neurocore

Spine Endoscopic Surgery for Disc Disease (**SENSE-D**): consensus-based guidelines

Round 2: comprehensive report

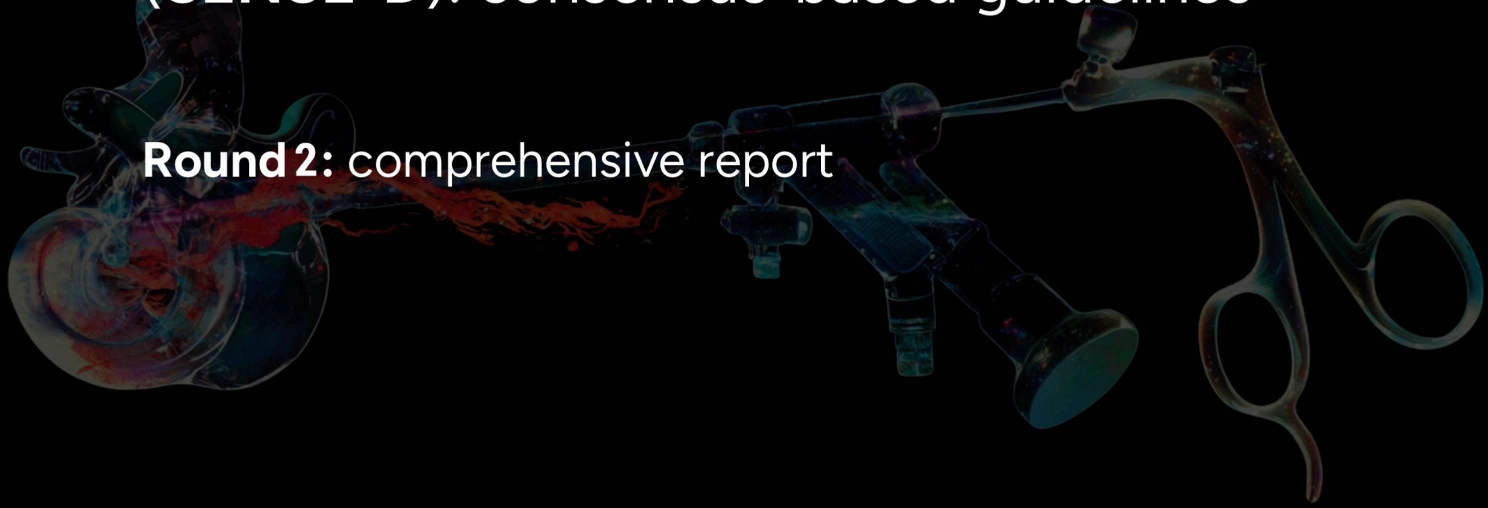

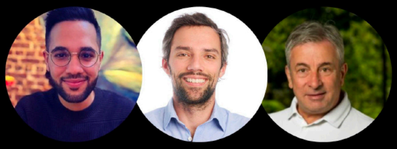

Mejdeddine Al Barajraji, MD
Richard Assaker, MD, PhD
Thibault Remacle, MD, PhD

Sciense
mejdi@sciense.org
447 Broadway, 2nd Floor
New York, NY 10013

This open report details results and additional propositions from each round, ensuring transparency and informing subsequent rounds.

Inclusion: endorsed by ≥75% of participants are immediately added to the core outcome set (COS) ✅

Exclusion: with <50% endorsement are removed—a call can be made by any participant ❌

Reevaluation: with more than 50 and less than 75% endorsement are reconsidered in the next round 🔄

Note that propositions

- are submitted for consideration a maximum of two rounds.
- not meeting COS criteria may still inform fork projects of dedicated working groups.

Concerning our treatment of additional propositions or insights, 'ADD-ONS,' they can be either:

- directly added to the next round ➕
- covered or integrated with others ⬇️
- considered valuable insights, not forming direct propositions 💡

Sciense
mejdi@sciense.org
447 Broadway, 2nd Floor
New York, NY 10013

I. Baseline items

Which patient variables should be systematically reported in studies mentioning EDDS?

<u>Demographics:</u>

58 responses

drug use	55% ❌	32 responses
tobacco consumption	74% ✅	43 responses
socioeconomic status	52% ❌	30 responses
+ family history of disc herniation	45% ❌	26 responses
+ recreational/sports activities	62% 🔄	36 responses
no opinion	9%	5 responses

ADD-ONS

- None

<u>Comorbidities:</u>

58 responses

cardiovascular diseases	66% ✅	38 responses
active infections	71% ✅	41 responses
+ urological disease	24% ❌	14 responses
+ occupational accident	41% ❌	24 responses
+ COVID infection	5% ❌	3 responses
+ osteoporosis	59% 🔄	34 responses
no opinion	3%	2 responses

ADD-ONS

- None

<u>Initial complaint:</u>

58 responses

neck or back stiffness	81% ✅	47 responses
+ night pain	69% 🔄	40 responses
no opinion	14%	8 responses

ADD-ONS

- Radicular symptoms

neurocore

Severity assessment:

77 responses

Morphological (imaging-based)	79% ✅	61 responses
Clinical Severity Scoring Systems	79% ✅	61 responses

↓

58 responses

+ disc height	86% ✅	50 responses
+ Meyerding grading (if listhesis)	83% ✅	48 responses
no opinion	2%	1 response

ADD-ONS

- Modic changes

Ongoing treatment(s):

58 responses

Acetaminophen (Paracetamol)	74% ✅	43 responses
Muscle relaxants	67% ✅	39 responses
Pain modulation: implantable devices	69% ✅	40 responses
no opinion	12%	7 responses

ADD-ONS

- None

neurocore

II. Practice items

Which practices should be systematically defined in studies reporting EDDS?

Clinical aspects:

Duration of symptoms prior to surgery ···

58 responses

+ weeks	59%	34 responses
✅ months	41%	24 responses
no opinion	0%	No responses yet

N.B : « months » already included in round 1, comparative reevaluation of "weeks" sub-item

Trial of conservative therapy prior to surgery	86% ✅	66 responses

↓

Medications prior to surgery

58 responses

yes	91% ✅	53 responses
no	4% ❌	2 responses
no opinion	5%	3 responses

Surgical aspects:

58 responses

patient positioning	83% ✅	48 responses
number of skin incisions	76% ✅	44 responses
size of skin incision(s)	48% ❌	28 responses
intraoperative imaging modalities used during the procedure	79% ✅	46 responses
use of a drill	74% ✅	43 responses
placement of a drain	66% ✅	38 responses
use of local haemostatic agents	59% ❌	34 responses
+ Drugs used during anesthesia	34% ❌	20 responses
+ Blood pressure levels during the procedure	40% ❌	23 responses
no opinion	5%	3 responses

neurocore

Technical aspects:

58 responses

Item	%	Status	Responses
Material Manufacturer and Model	64%	❌	37 responses
Endoscope Specifications	78%	✅	45 responses

Which one(s) ?
46 responses

Item	%	Status	Responses
Diameter	76%	✅	35 responses
Working channel size	76%	✅	35 responses
Field of view (e.g., 20°, 30°, or 70°)	85%	✅	39 responses
Illumination type (e.g., fiber-optic, LED)	39%	❌	18 responses

Item	%	Status	Responses
Instrumentation Used: Burrs/Drills	71%	✅	41 responses

Which one(s)?
42 responses

Item	%	Status	Responses
Drill type (e.g., diamond, carbide)	86%	✅	36 responses
Speed settings	38%	❌	16 responses
Diameter of the drill or burr	79%	✅	33 responses

Item	%	Status	Responses
Instrumentation Used: Radiofrequency or Laser Devices	66%	✅	38 responses

Which one(s) ?
38 responses

Item	%	Status	Responses
Settings used (e.g., power, frequency).	76%	✅	29 responses
Purpose (e.g., coagulation, annular modulation).	87%	✅	33 responses

Item	%	Status	Responses
Irrigation System	71%	✅	41 responses

Which one(s) ?
41 responses

Item	%	Status	Responses
Irrigation pump type and settings (e.g., flow rate, pressure).	88%	✅	36 responses
Type of irrigation fluid (e.g., saline, Ringer's lactate).	68%	✅	28 responses
Type of flow (e.g. fountain-like, stream-line)	61%	❌	25 responses
Epinephrine use in case of bleeding	61%	❌	25 responses
+ Pressure modulation according to surgical phases	56%	🔄	23 responses

Item	%	Responses
no opinion	3%	2 responses

Sciense
mejdi@sciense.org
447 Broadway, 2nd Floor
New York, NY 10013

neurocore

III. Outcome items

Which outcomes should be reported in a standardized way?

58 responses

Option	%		Responses
Pain Reduction: NRS	100%	✅	58 responses
Neurological Improvement: bladder/bowel function	67%	✅	39 responses
Neurological Improvement: Motor (MRC)	90%	✅	52 responses
Functional Improvement: Return to Work or Daily Activities (days)	90%	✅	52 responses
Length of Hospital Stay	91%	✅	53 responses
+ Work Productivity and Activity Impairment (WPAI) Questionnaire	40%	❌	23 responses
no opinion	0%		No responses yet

ADD-ONS

- None

Which complications should we report in a standardized way ?

58 responses

Option	%		Responses
Vascular injury	67%	✅	39 responses
Wrong-site surgery	64%	❌	37 responses
Persistent or Worsened Pain	97%	✅	56 responses
Failure to Complete Endoscopically (conversion)	91%	✅	53 responses
+ Neuropathic pain	84%	✅	49 responses
+ Return to OR within 30 days	86%	✅	50 responses
no opinion	2%		1 response

ADD-ONS

- None

Sciense
mejdi@sciense.org
447 Broadway, 2nd Floor
New York, NY 10013

IV. Perspectives

Which tools would be useful for spine surgeons regarding EDDS?

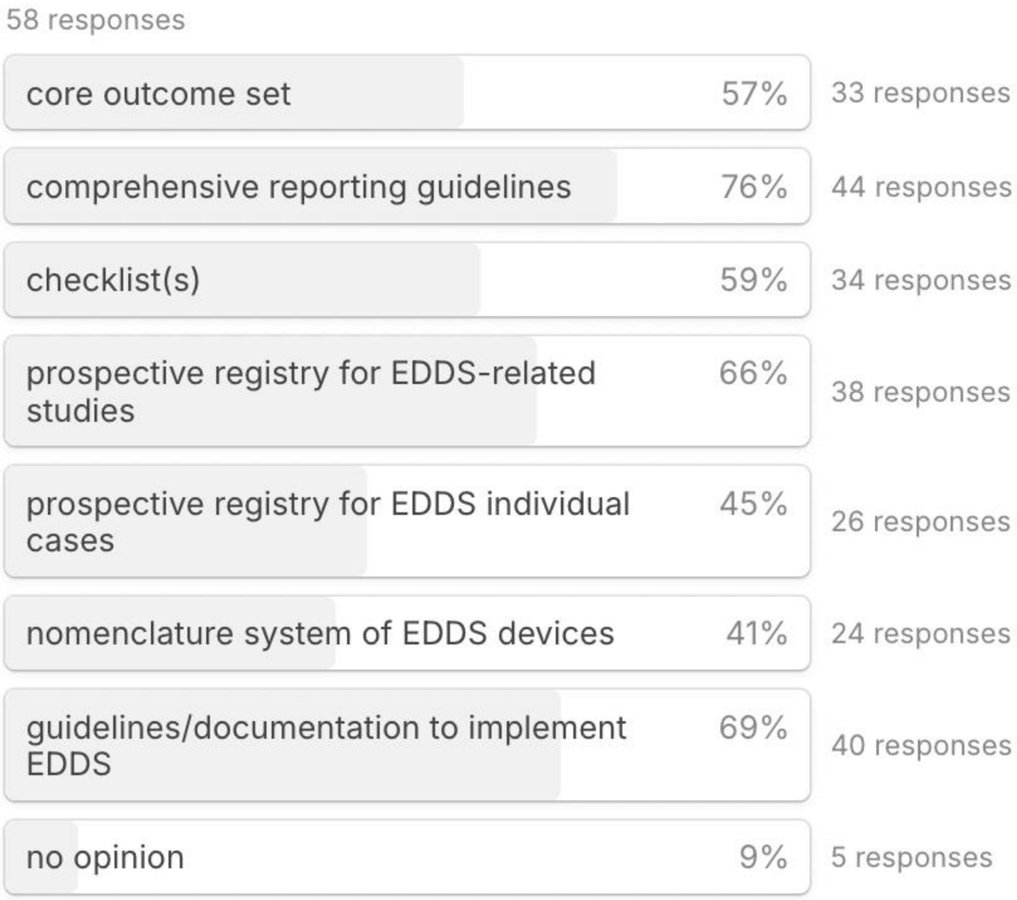

Supplement: Multimedia component 2 [file mmc2.pdf]
